# Chemo-bio catalysis using carbon supports: application in H_2_-driven cofactor recycling†

**DOI:** 10.1039/d1sc00295c

**Published:** 2021-05-07

**Authors:** Xu Zhao, Sarah E. Cleary, Ceren Zor, Nicole Grobert, Holly A. Reeve, Kylie A. Vincent

**Affiliations:** Department of Chemistry, University of Oxford, Inorganic Chemistry Laboratory South Parks Road Oxford OX1 3QR UK holly.reeve@chem.ox.ac.uk kylie.vincent@chem.ox.ac.uk; Department of Materials, University of Oxford Parks Road Oxford OX1 3PH UK nicole.grobert@materials.ox.ac.uk

## Abstract

Heterogeneous biocatalytic hydrogenation is an attractive strategy for clean, enantioselective C=X reduction. This approach relies on enzymes powered by H_2_-driven NADH recycling. Commercially available carbon-supported metal (metal/C) catalysts are investigated here for direct H_2_-driven NAD^+^ reduction. Selected metal/C catalysts are then used for H_2_ oxidation with electrons transferred *via* the conductive carbon support material to an adsorbed enzyme for NAD^+^ reduction. These chemo-bio catalysts show improved activity and selectivity for generating bioactive NADH under ambient reaction conditions compared to metal/C catalysts. The metal/C catalysts and carbon support materials (all activated carbon or carbon black) are characterised to probe which properties potentially influence catalyst activity. The optimised chemo-bio catalysts are then used to supply NADH to an alcohol dehydrogenase for enantioselective (>99% ee) ketone reductions, leading to high cofactor turnover numbers and Pd and NAD^+^ reductase activities of 441 h^−1^ and 2347 h^−1^, respectively. This method demonstrates a new way of combining chemo- and biocatalysis on carbon supports, highlighted here for selective hydrogenation reactions.

## Abstract 2

Heterogeneous chemo-bio catalytic hydrogenation is an attractive strategy for clean, enantioselective C=X reduction.

## Graphical abstract

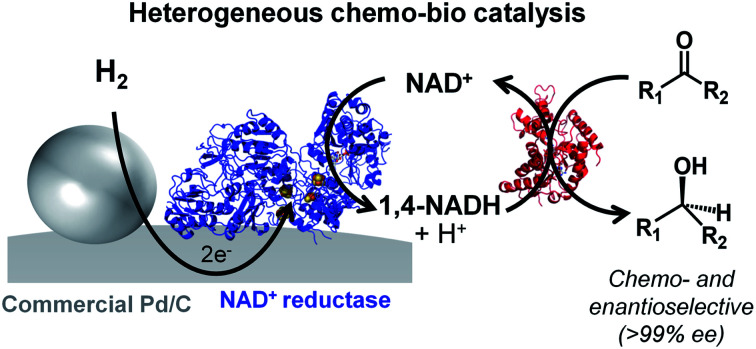

## 1. Introduction

The ability to combine metal- and enzyme-catalysed steps in one-pot reactions simplifies synthetic approaches that rely upon both chemo- and biocatalysis.^1–3^ Common challenges associated with chemo-bio systems are reaction condition compatibility (*e.g.* solvent, pH, temperature, pressure) and mutual poisoning of metal and enzyme.^4–7^ Optimisation of catalyst preparation and reaction conditions are therefore required to ensure both metal and enzyme catalysts are simultaneously active.^8^ Reaction times and processing steps are often minimised when both catalysts are immobilised on heterogeneous supports,^9^ therefore the known benefits of chemo-bio catalysis could be amplified by co-immobilising the two catalysts onto a heterogeneous support.

Many synthetically useful biocatalysed transformations depend on redox cofactors.^10^ One of the most common cofactors, NADH, acts as a hydride source for enantio- and chemoselective reductions, but its stoichiometric use is prohibitively expensive when reactions are carried out on a manufacturing scale. Therefore, continual recycling of a catalytic quantity of NAD^+^/NADH is necessary for these reactions to be industrially viable, and current practices rely on enzymatic cofactor recycling driven by superstoichiometric reductants (*e.g.* glucose) leading to a build-up of waste.^11^ Clean, highly atom-economical H_2_-driven NADH generation has been reported using whole cell systems,^12^ isolated enzymes,^13^ and heterogeneous biocatalytic strategies.^14,15^ The enzymes contain an H_2_ oxidation site and an NAD^+^ reduction site that are in direct electronic contact, either natively or provided by co-immobilisation on an electronically conducting carbon support. These methods are selective for producing bioactive NADH and have been demonstrated with a range of C=X bond-reducing enzymes.^14,16,17^

Organometallic catalysts have also been reported for mediated H_2_-driven NAD^+^ reduction. Early examples relied on H_2_-driven reduction of electron carriers (*e.g.* pyruvate, Safranine) that went on to reduce NAD(P)^+^.^18,19^ More recently, homogeneous organometallic complexes have been used to produce NADH under H_2_ without the need for mediators,^20^ though these complexes are known to face biocompatibility issues when used in one-pot reactions with enzymes that consume NADH (*e.g.* alcohol dehydrogenases).^6,21^

**Fig. 1:**
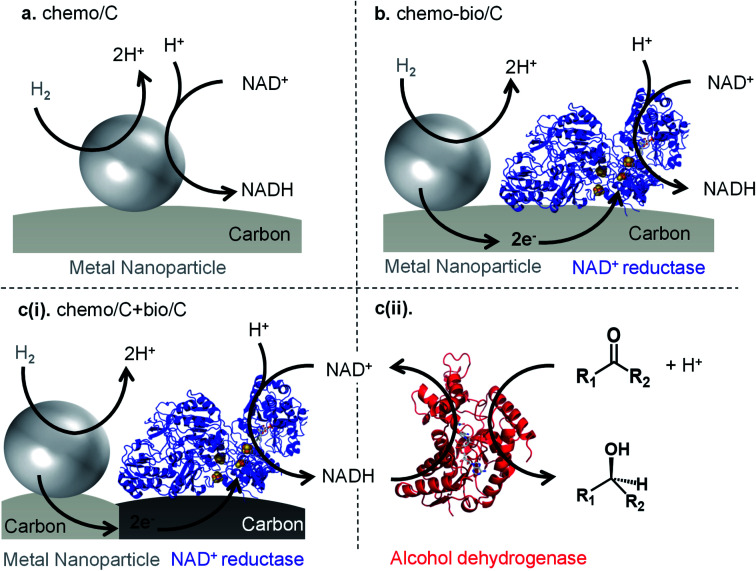
Methods for H_2_-driven NADH generation using carbon-supported metal nanoparticles. (a) Both H_2_ oxidation and NAD^+^ reduction must occur at metal “chemo/C′′ site. (b) Metal-catalysed H_2_ oxidation is coupled to a co-immobilised NAD^+^ reductase for “chemo-bio/C′′ catalysed NADH generation. (c)(i) Mixed carbon agglomerate “chemo/C+bio/C′′ catalyst involves mixing separate carbon-supported metal and carbon-supported NAD^+^ reductase, which then agglomerate such that electron transfer is still possible between the site-separated catalysts (see later). (c)(ii) H_2_-driven cofactor generation can extend to NADH-dependent biocatalysis (*e.g.* asymmetric ketone reduction by an alcohol dehydrogenase, “ADH”).

Wang and co-workers have reported platinum nanoparticles (NPs) supported on carbon or metal oxides for H_2_-driven NAD^+^ reduction (Fig. 1a).^22,23^ Elevated temperatures and pressures were used to overcome the activation energy for Pt-catalysed NAD^+^ reduction, but side-products resulted (*e.g.* from over-reduction of the pyridine ring) in addition to the desired 1,4-NADH. Under milder conditions used to supply an alcohol dehydrogenase with 1,4-NADH, only seven cofactor turnovers occurred after 100 h of reaction time, suggesting that further catalyst development would be beneficial.^22^

Carbon materials (typically graphite, carbon black and activated carbon) are widely used as supports for metal NPs,^24–26^ and have emerged as versatile supports for enzymes.^27–29^ These carbon supports are cheap, available in a range of forms, and offer pH robustness, electron transfer, and high surface area compared with other supports for metal and enzyme immobilisation (*e.g.* silica or alumina).^30^ We therefore wondered if carbon particles could act as a support for hybridising chemo- and biocatalysis onto a single heterogeneous support.

Herein, we investigate a range of catalyst systems for H_2_-driven NAD^+^ reduction (Fig. 1). We first evaluate commercial metal on carbon “chemo/C” catalysts, then these catalysts are also used in the preparation of “chemo-bio/C′′ catalysts through simple adsorption of an NAD^+^ reducing enzyme onto the chemo/C (Fig. 1b). We have previously shown that a conductive carbon support transfers electrons from a H_2_-oxidising enzyme to a co-immobilised NAD^+^ reductase,^15,31^ so we hypothesised that the same should be possible from a metal nanoparticle capable of H_2_ oxidation to the co-immobilised NAD^+^ reductase enzyme. The NAD^+^ reductase contains a flavin mononucleotide active site which takes up two electrons *via* an electron-relay chain of iron-sulphur clusters in the protein. A proton from solution then combines with the two electrons at the flavin, and the resulting hydride is transferred to NAD^+^ to selectively form, exclusively, the biologically active 1,4-NADH (**2**). Additionally, a mixed carbon agglomerate “chemo/C+bio/C′′ catalyst is developed in which the metal and enzyme are each immobilised on separate carbons (Fig. 1c(i)). When mixed together, the agglomerated carbons should still allow electron transfer from metal to enzyme *via* the carbon (see later). Each catalyst is compared for activity and selectivity for producing biologically active 1,4-NADH (**2**) using a range of characterisation techniques, and the different commercial carbon materials are analysed and compared. Both the chemo-bio/C and the chemo/C+bio/C catalyst types are then paired with an NADH-dependent alcohol dehydrogenase to promote asymmetric ketone reductions with high selectivity and turnover numbers (Fig. 1c(ii)).

## 2. Results and discussion

### 2.1 Screening of chemo/C catalysts for H_2_-driven NADH generation

**Fig. 2:**
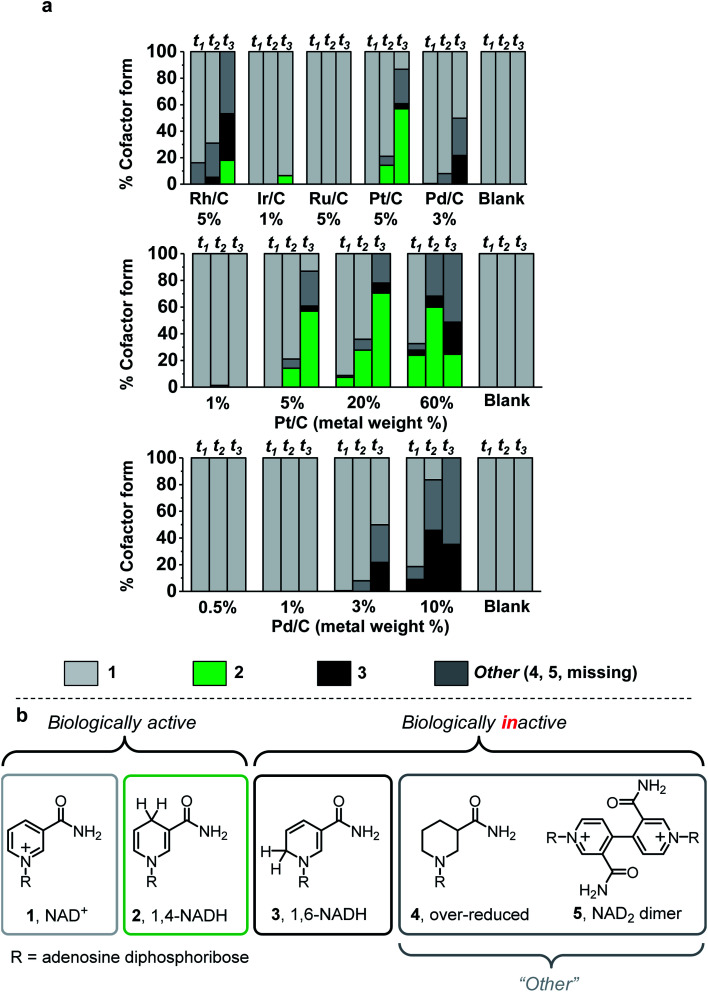
Cofactor compositions after H_2_-driven NAD^+^ reduction by different chemo/C catalysts. a. Values were calculated using a combination of quantitative ^1^H NMR spectroscopy and UV-visible spectroscopy at three time points (*t*_1_ = 30 min, *t*_2_ = 2 h, *t*_3_ = 17 h). At 17 h, 5–8% relative standard deviation was determined from reactions performed in triplicate using 3 wt% Pd/C (Fig. S1, ESI†). Reaction conditions: 2 mL of 5 mM NAD^+^ in Tris–HCl (50 mM, pH 8.0) was stirred with 0.32 mg commercial chemo/C under a steady flow of H_2_ (1 bar) at room temperature. “Blank” experiments were run as a control in the absence of catalyst, and our previous report showed that carbon black (BP2000) does not catalyse H_2_-driven NAD^+^ reduction.^14^ Further details in ESI (S1.5.1†). (b) Simplified structures of biologically active and inactive nicotinamide cofactors.

Five commercial metals (rhodium, iridium, ruthenium, platinum, and palladium) immobilised on activated carbon and carbon black with low metal loading (1–5 wt%) were evaluated for NADH production under the reaction protocol described in S1.5.1 (see ESI for method and Table S1† for details on the carbon material type of each catalyst). The figures of merit used to evaluate chemo/C catalysts were activity and selectivity for generating bioactive NADH, while avoiding metal-catalysed cofactor degradation over time.^32^ The reaction course was monitored by taking aliquots for analysis at 30 min, 2 h, and 17 h time points (*t*_1_, *t*_2_, and *t*_3_, Fig. 2a). Cofactor composition of unreacted NAD^+^ (**1**, light grey), biologically active 1,4-NADH (**2**, green) and undesired 1,6-NADH (**3**, black)^33^ was quantified using ^1^H NMR spectroscopy. Simplified cofactor structures are shown in Fig. 2b. UV-vis spectroscopy and HPLC were used to verify product composition (ESI Fig. S1–S3 provide analytical details†). The “other” category (dark grey) encompasses cofactor forms that are not easily quantified by NMR spectroscopy (*e.g.***4**,^22^**5**, Fig. S3†) as well as cofactor missing from the sum of all other categories. “Missing” cofactor could be due to adsorption onto the carbon material. This adsorption was suggested by a control experiment in which a decrease in concentration of **2** was quantified when stirred with the catalysts under N_2_ (Fig. S4†).

Under these reaction conditions, Rh/C (5 wt%) showed high conversion but poor selectivity, while Ir/C (1 wt%) showed selectivity for **2**, but low activity. Ru/C (5 wt%) was inactive toward NAD^+^ reduction. Pt/C (5 wt%) exhibited reasonable conversion rates and fairly high selectivity for **2**, while Pd/C (3 wt%) did not produce any detectable amount of the desired product, **2**, at any time point.

To determine if metal wt% influences NAD^+^ reduction selectivity, we chose Pt/C and Pd/C (0.5–60 wt%) for further investigation based on the commercial availability of these metals at a range of loadings. The majority of the Pt/C and Pd/C catalysts were supplied in powder form, however 20 and 60 wt% Pt/C and 0.5 wt% Pd/C were supplied as mm-cm carbon particles. In order to achieve reactions with comparable catalyst handling, the large particles were ground to a paste in water using a mortar and pestle, then left in a 60 °C oven overnight before use. Reactions were again set up following the procedure described in S1.5.1,† and the same analytical techniques were used for evaluation of cofactor composition.

When 5–60 wt% Pt/C were used, some preference for **2** was observed in the first 2 h, however other cofactor forms were detected after 17 h (Fig. 2a). This observation suggests that onward reaction of **2** with the metal catalysts can lead to by-products, confirmed by control experiments in which chemo/C catalysts were stirred in a solution of **2** under H_2_ resulting in production of further products (*e.g.***3**, Fig. S5†). Only trace conversion was observed when 1 wt% Pt/C was used. For comparison, Wang *et al.* reported that 1 wt% Pt/C can reduce **1** under more forcing reaction conditions (10 bar H_2_, 37 °C, pH 7), and that this provided a mixture of unknown side products in addition to **2** and **3**.^23^

At no loading did the Pd/C catalysts produce a detectable quantity of **2**. The 3 wt% and 10 wt% Pd/C predominately produced inactive cofactor at every time point, which precludes these catalysts from use in cofactor recycling. Low metal loadings of Pd (0.5–1 wt%) did not appear to act on **1** and did not generate any inactive cofactor forms. We therefore took these low Pd content catalysts for characterisation of the carbon supports, then tested if they could be coupled with an immobilised NAD^+^ reductase where electrons from Pd-catalysed H_2_-oxidation could be supplied to the NAD^+^ reductase for selective, enzymatic reduction of **1**.

### 2.2 Characterisation of selected chemo/C materials

**Fig. 3:**
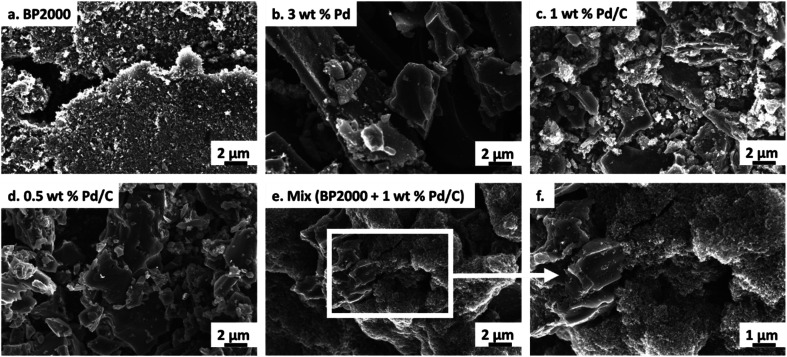
SEM micrographs showing morphology of carbon supports. (a) BP2000 has uniform morphology with particle sizes in the nm range. (b–d) Pd/C have small particles as well as large agglomerates and micron size platelets. (e–f) The mix of {BP2000 + 1 wt% Pd/C} shows large agglomerates and platelets (likely Pd/C) that appear to be coated with small spherical particles (likely BP2000, see additional image in Fig. S9, ESI†).

The commercial 0.5–3% Pd/C were composed of differing carbon types (carbon black or activated carbon, see Table S1; † differing morphologies are evident in SEM images, Fig. 3 and X-ray powder diffraction patterns, Fig. S6†). We sought to understand the variability of the carbon support materials in more detail. In particular, we studied the carbon properties that might impact enzyme adsorption (degree of graphitisation, surface area, pore diameter, uniformity, elemental surface composition)^34–36^ using a variety of techniques as a way to evaluate the chemo/C catalysts for their potential use in chemo-bio/C catalysts. A metal-free, highly porous carbon black nanopowder (BP2000), which has previously been used as a carbon support for biocatalytic H_2_-driven NADH generation,^14,16^ was used for comparison.

All of the Pd/C carbons and BP2000 showed low degrees of graphitisation by Raman spectroscopy (Fig. S7†). Nitrogen adsorption analysis showed BP2000 has over 50% more surface area compared with the other carbon supports (≥696 m^2^ per g according to Langmuir model) and the largest average pore size (5.6 nm, Table S4†). SEM images of the carbon samples show that BP2000 has the most uniform morphology (Fig. 3a) whereas the Pd/C samples had some small particles, large agglomerates and platelets (Fig. 3b–d). Some or all of these properties likely improve enzyme adsorption: Bradford assays reveal only 3% enzyme left in the supernatant after NAD^+^ reductase was immobilised onto BP2000. This value was ≥20-fold lower than the Pd/C (Table S5†), demonstrating improved adsorption on BP2000. Table S5† summarizes EDX elemental analysis of the material surfaces which did not reveal any correlation between elemental composition and protein adsorption, although tuning of surface chemistry through modification of the support is something that could be explored in the future.

### 2.3 Co-immobilised Pd and NAD^+^ reductase “chemo-bio/C” catalysts

Commercially available Pd/C catalysts (0.5–3 wt%) were selected to act as both the “chemo” H_2_-oxidation site and carbon support for the preparation of chemo-bio/C catalysts for generating NADH, and the “bio” catalysts were a co-immobilised NAD^+^ reductase. Palladium was chosen over platinum due to wider commercial range of <5 wt% metal loadings. The chemo-bio/C catalysts were straightforwardly prepared on the benchtop by incubating NAD^+^ reductase (various loadings, from *R. eutropha*, 170 kDa)^14,37^ with a slurry of Pd/C (100 μg in 5 μL Tris–HCl buffer) under N_2_ over ice in a stirred round bottom flask for 1 h. Control experiments confirmed that NAD^+^ reductase has negligible activity for NAD^+^ reduction under H_2_ without the presence of metal (Fig. S10†), and we have previously confirmed that this enzyme exclusively produces **2** in an NMR spectroscopy study.^16^

**Fig. 4:**
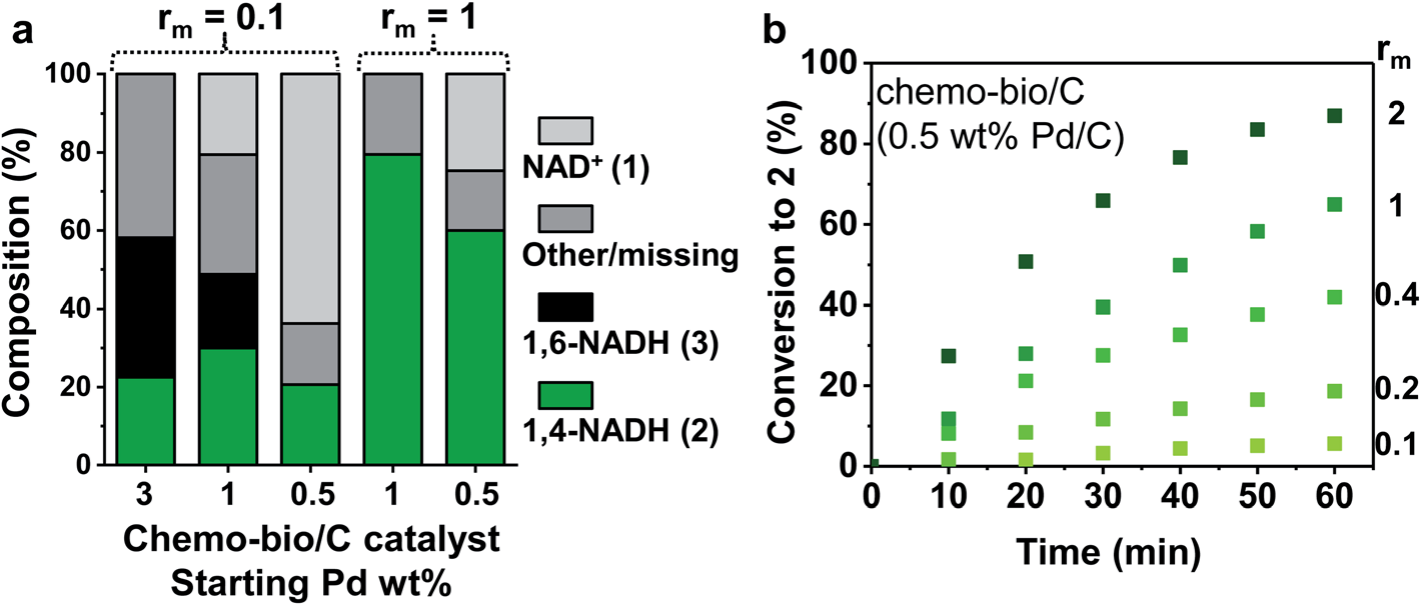
Reaction progress of chemo-bio/C catalysts using different Pd and enzyme loadings. (a) Cofactor compositions after 17 h determined by ^1^H NMR spectroscopy. Reaction conditions: 1 mM NAD^+^ stirred with chemo-bio/C catalysts formed from 100 μg Pd/C and the corresponding mass of enzyme following procedure from S1.5.3 (see detail in ESI†). (b) Conversion to **2** over 60 min monitored by absorbance at 340 nm using *in situ* UV-visible spectroscopy. Reaction conditions (see further details in S1.5.2†): 0.1 mM NAD^+^ mixed with chemo-bio/C catalysts formed from 20 μg 0.5 wt% Pd/C and enzyme loadings ranging from 2–40 μg (giving an *r*_m_ range of 0.1–2).

The mass ratio of enzyme and carbon is defined as 
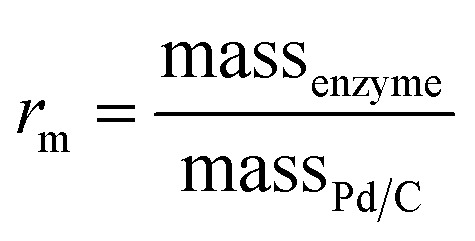
, which was initially set to 0.1. Chemo-bio/C catalysts were evaluated for NADH generation activity and selectivity using ^1^H NMR spectroscopy following the procedure described in S1.5.3† (Fig. 4a).

The 3 wt% Pd chemo-bio/C catalyst produced some biologically active reduced cofactor, **2**, however significant formation of side products highlights the activity of the metal at this loading toward formation of inactive cofactor forms, precluding use of this chemo-bio/C catalyst for cofactor recycling.

Whereas the 1% Pd/C chemo/C catalyst alone showed no activity for reduction of **1** (Fig. 2a), on incorporation of NAD^+^ reductase the chemo-bio/C catalyst showed formation of **2** (Fig. 4a). The formation of **3** as by-product could be from Pd reacting with **1** or enzyme-produced **2**. For the 0.5% Pd chemo-bio/C, selectivity for **2** was observed. In further experiments, the NAD^+^ reductase loading was increased to achieve *r*_m_ = 1, leading to selective generation of the desired product, **2**, with both 0.5 and 1% Pd chemo-bio/C. In these experiments, no inactive cofactor was detected, although there is some ‘missing’ cofactor, which could be due to adsorption onto the carbon support.

Noting that the Pd/C catalysts alone did not produce detectable quantities of **2** at any metal loading (Fig. 2a), the fact that **2** is observed as a major product in the reactions in which the NAD^+^ reductase is co-immobilised on Pd/C materials to form the chemo-bio/C catalysts indicates that the NAD^+^ reductase must be responsible for the NAD^+^ reduction here. Since the NAD^+^ reductase cannot oxidise H_2_ itself, these results are indicative of electron transfer through the carbon from H_2_ oxidation at Pd to the co-immobilised NAD^+^ reductase enzyme.

We examined more closely the impact of *r*_m_ on conversion to **2**. Different masses of NAD^+^ reductase were incubated with a slurry of 0.5 wt% Pd/C, then the chemo-bio/C catalysts were assayed for NAD^+^ reduction activity by *in situ* UV-visible spectroscopy following the method described in S1.5.2.† Conversion and enzyme turnover frequency (TOF, mol **2** per mol NAD^+^ reductase per hour) were compared (Fig. 4b and Table 1). Palladium TOF (mol **2** per mol Pd per hour) was also calculated under the assumption that each equivalent of NADH generated requires one equivalent of H_2_ to be oxidised by Pd. When entry 4 was repeated in triplicate, a relative standard deviation of 7% was calculated for metal and enzyme TOF, showing good reproducibility.

**Table 1:** Catalyst activities in H_2_-driven NAD^+^ reduction at varying quantities of NAD^+^ reductase (immobilised on 0.5 wt% Pd/C, 20 μg) and varying [**1**]^a^

Entry	*r*_m_	NAD^+^ reductase loading (μg)	[**1**] (mM)	NAD^+^ reductase TOF^b^ (h^−1^)	Pd TOF (h^−1^)
1	0.1	2	0.1	530	6.6
2	0.2	4	0.1	785	20
3	0.4	8	0.1	979	49
4	1	20	0.1	520	65
5	2	40	0.1	449	113
6^c^	2	40	0.5	877	218
7^c^	2	40	5	n.d.^d^	n.d.^d^
8^e^	1	20	0.1	143	17
9^e^	2	40	0.1	316	78

a Reaction conditions follow S1.5.2 with chemo-bio/C catalysts in entries 1–5 formed from 20 μg 0.5 wt% Pd/C and the indicated enzyme loadings. When entry 4 was repeated in triplicate, a relative standard deviation of 7% was calculated for the TOFs.

b Activity was measured during the linear phase of the reaction.

c Entries 6 and 7 followed S1.5.3 in a stirred round bottomed flask.

d Not determined.

e Entries 8–9 were carried out following S1.5.2 inside a glovebox and the reaction was mixed using a magnetic stirrer bead using chemo/C+bio/C catalysts 
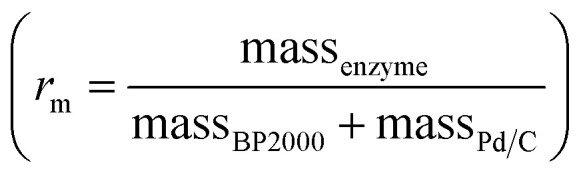
 formed from 10 μg BP2000, 10 μg 1 wt% Pd/C and the indicated enzyme loadings.

Using a standard 0.1 mM **1**, palladium activity improved with increased enzyme loading (entries 1–5, Table 1) with the highest Pd TOF (113 h^−1^) calculated at the highest NAD^+^ reductase loading (entry 5). These activities exceed other reported metal-catalysed H_2_-driven^20,22^ and formate-driven NADH-generation under ambient conditions by 3–45-fold.^38–40^ The *r*_m_ = 0.4 catalyst provided the highest NAD^+^ reductase activity (979 h^−1^; 96 nmol min^−1^ mg^−1^, entry 3). The catalysts comprising higher *r*_m_ had lower enzyme activities, possibly due to limitations of enzyme adsorption (Table S6†), or because the chemo-bio/C catalyst becomes H_2_ oxidation rate-limited.

The use of 0.5 mM **1** (entry 6) led to higher enzyme activity (877 h^−1^; 86 nmol min^−1^ mg^−1^) and Pd TOF (218 h^−1^). At 5 mM **1** the reaction conversion plateaued and only 6% conversion was reached after 5 h (entry 7). The Pd was seemingly inhibited by the high cofactor concentration.^20,41^ A control experiment (Fig. S11†) supports this hypothesis: when the chemo-bio/C was stirred in a solution of 4 mM **1** under H_2_ which was then diluted to 0.5 mM using Tris–HCl buffer, the catalyst activity also plateaued rather than duplicate the 0.5 mM conversion rate. In a cofactor recycling application, lower NAD^+^ loadings would be used in order to keep reaction cost and waste low, hence this limitation was not considered a setback.

### 2.4 Mixed carbon agglomerate “chemo/C+bio/C” catalyst design

In previous studies, we have observed excellent adsorption of enzymes onto BP2000.^29^ This inspired us to develop a mixed carbon agglomerate chemo/C+bio/C catalyst composed of NAD^+^ reductase on BP2000 that was then mixed with Pd/C, with electron transfer expected to occur *via* the carbon agglomerates (Fig. 1c(i)). This strategy also enabled us to test if keeping the enzyme and metal spatially separated alleviated any mutual inhibition. SEM images showed that the small, spherical BP2000 (Fig. 3a) coats the larger Pd/C platelets and agglomerates (Fig. 3c) when the two are mixed as a slurry (Fig. 3e, f and S9†).

After mixing one part {1 wt% Pd/C} with one part {BP2000 modified with NAD^+^ reductase}, we confirmed that the surface palladium composition corresponded with that of commercial 0.5 wt% Pd/C using SEM/EDX (1.4% and 1.5%, respectively; Table S5†). This enabled us to directly compare the chemo/C+bio/C catalyst activities to the equivalent 0.5 wt% chemo-bio/C catalysts (entries 8–9, Table 1). The mass ratio of enzyme to carbon 
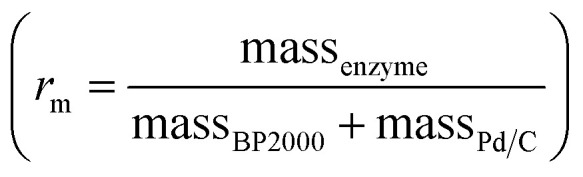
 was set to 1 and 2. *In situ* UV-visible spectroscopy was used to calculate activity following the procedure described in S.1.5.2.† Product composition was determined using ^1^H NMR spectroscopy in a separate experiment where 1 mM NAD^+^ was used following the method described in S.1.5.3, which confirmed selectivity for **2** (Fig. S12†).

Compared with the co-immobilised chemo-bio/C catalysts, the chemo/C+bio/C had lower activities per mg enzyme (in Table 1, compare entries 4 and 8; 5 and 9). The best Pd TOF (78.0 h^−1^, *r*_m_ = 2) was lower than the TOF achieved using the corresponding chemo-bio/C catalyst (113 h^−1^). One explanation could be due to fouling of the palladium by BP2000, thus less metal exposure for H_2_ oxidation. In an alternative preparation method, the BP2000 and Pd/C were mixed prior to enzyme adsorption. When *r*_m_ = 1, there was a longer lag time for activity, after which the NAD^+^ reductase was comparable (163 h^−1^; 16 nmol min^−1^ mg^−1^, Fig. S13†).

The observation of activity with the chemo/C+bio/C catalysts supports our hypothesis that there is good physical contact between the two carbon types, facilitating electron transfer between metal and enzyme. Furthermore, the use of BP2000 for the mixed carbon platform in principle enables chemo-bio catalysts to be prepared from chemo/C catalysts that do not themselves efficiently adsorb enzymes. It also permits the user to adjust metal wt% in order to tune reaction selectivity. Finally, although activity was not improved here, the ability to physically separate the enzyme and metal NP may circumvent mutual inactivation in catalysts for which this is an issue.

### 2.5 Asymmetric ketone reductions by chemo-bio H_2_-driven cofactor recycling

The chemo-bio catalysts were used to continually supply **2** to an (*S*)-selective alcohol dehydrogenase (ADH-105, Johnson Matthey) for acetophenone (**6**) reduction (Fig. 1c(ii)). Reactions were carried out following the procedure described in S.1.5.4† and analysed for conversion and enantiomeric excess (>99% ee in all cases) by chiral phase GC-FID at 2 h and 24 h (see Fig. S14†). Pressurised reactions (4 bar H_2_) were performed in a pressure vessel, and atmospheric H_2_ (1 bar) was achieved by a steady flow of gas over the reaction headspace.

Initial experiments were designed to optimise parameters for high conversion to **7** (Table 2). Chemo-bio/C catalysts formed from 1 wt% Pd/C promoted higher conversions compared with those formed from 0.5 wt% Pd/C (entries 1–4). Entry 6 shows that a comparable conversion was obtained when the analogous chemo/C+bio/C catalyst was used (compare to entry 3). We observed enhanced dispersion of the heterogeneous chemo/C+bio/C catalyst, which remained as a slurry with agitation, compared with the chemo-bio/C which settled to the bottom of the reaction tube. To avoid limitations due to mixing, we used the chemo/C+bio/C to further explore reaction parameters (entries 6–10). Following this optimisation, we returned to the chemo-bio/C using the optimal conditions with success (97% conversion of 20 mM **6** under ambient H_2_, entry 5).

**Table 2:** Optimisation of H_2_-driven chemo-bio acetophenone reduction^a^

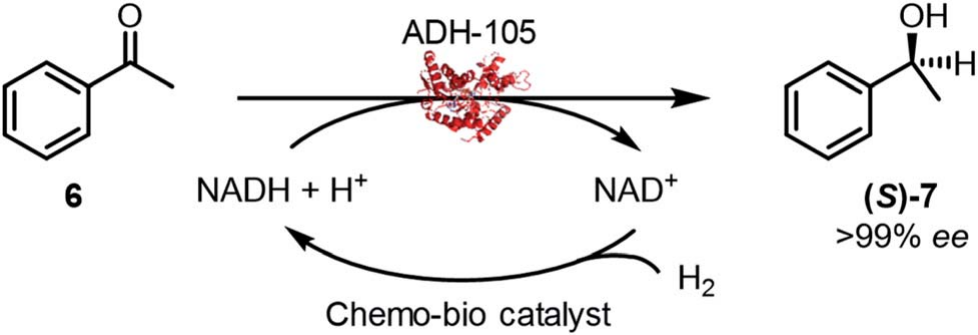								
Entry	*P*_H_2__ (bar)	[NAD^+^] (mM)	[**6**] (mM)	Chemo-bio catalyst			ADH (mg mL^−1^)	Conversion (%)
				Type	Pd wt%	mg mL^−1^		
1	4	0.5	5	Chemo-bio/C, *r*_m_ = 1	0.5	0.3	0.2	19
2	4	0.5	20	Chemo-bio/C, *r*_m_ = 1	0.5	0.3	0.2	2.4
3	4	0.5	5	Chemo-bio/C, *r*_m_ = 1	1	0.3	0.2	41
4	4	0.5	20	Chemo-bio/C, *r*_m_ = 1	1	0.3	0.2	15
**5**	**1**	**0.1**	**20**	**Chemo-bio/C, *r***_**m**_ **= 1**	**1**	**0.8**	**0.4**	**97**
6	4	0.5	5	Chemo/C+bio/C, *r*_m_ = 1	1	0.2	0.2	46
7	4	0.1	5	Chemo/C+bio/C, *r*_m_ = 1	1	0.4	0.4	>99
**8**	**4**	**0.1**	**20**	**Chemo/C+bio/C, *r***_**m**_ **= 1**	**1**	**0.4**	**0.4**	**93**
9	1	0.1	20	Chemo/C+bio/C, *r*_m_ = 2	1	0.8	0.4	98
**10**	**1**	**0.05**	**20**	**Chemo/C+bio/C, *r***_**m**_ **= 2**	**1**	**0.8**	**0.4**	**96**
11	4	0.1	20	Unmodified Pd/C	1	0.1	n/a	3 (racemic)

a Reactions were mixed for 24 h in H_2_-saturated buffer solution (50 mM Tris–HCl, pH 8.0) that contained NAD^+^, **6**, and 1 vol% DMSO following S1.5.4. Conversions were calculated after extracting reaction mixture into EtOAc using chiral-phase GC-FID. Entries 1–4, 6–8 and 11 : 1 mL reaction volume mixed in a pressure vessel that was oscillated at 26 rpm. Entries 5, 9–10 : 0.5 mL reaction volume mixed under a flow of H_2_ by shaking at 500 rpm. A standard deviation of 1% conversion was calculated when entry 8 was performed in triplicate. Rows in bold represent experiments also highlighted in Table 3.

A control experiment in which 1 wt% Pd/C was used under 4 bar H_2_ resulted in 3% conversion to racemic **7** (entry 11), which aligns with prior reported direct hydrogenation of **6** to racemic **7** by 10 wt% Pd/C in water.^42^ Since **(R)-7** was never detected when our chemo-bio catalysts were used with ADH, any background non-enzymatic reduction of **6** directly by Pd is negligible.

While we were pleased that both types of chemo-bio catalysts could be used with ADH-105 to provide high conversions of **6**, the chemo/C+bio/C catalyst tended give better catalyst figures of merit (summarised in Table 3). When 4 bar H_2_ was used with chemo/C+bio/C, the highest Pd turnover frequency (TOF, 441 h^−1^) and NAD^+^ reductase TOF (2347 h^−1^, not tabulated) were achieved (entry 2), which could be a result of improved H_2_ availability. This NAD^+^ reductase TOF is on the same order of magnitude as that achieved when a hydrogenase was used in place of palladium under ambient pressure (3168 h^−1^),^14^ which suggests that Pd does not significantly inhibit NAD^+^ reductase activity.

**Table 3:** Examples showcasing different figures of merit for chemo-bio acetophenone reduction^a^

Entry	*P*_H_2__ (bar)	Chemo-bio catalyst		Conversion^b^ (%)	Pd TOF^c^ (h^−1^)	NAD^+^ TN^b^	TTN Pd^b^	TTN NAD^+^ reductase^b^
		Type	mg mL^−1^					
1	1	Chemo-bio/C, *r*_m_ = 1	0.8	97	38	194	780	8400
2	4	Chemo/C+bio/C, *r*_m_ = 1	0.4	93	**441**	186	2640	**14 000**
3^d^	1	Chemo/C+bio/C, *r*_m_ = 2	0.8	96	136	**384**	2960	8300
4^e^	1	Chemo/C+bio/C, *r*_m_ = 2	0.8	55	215	220	**3390**	9500

a Reactions were mixed for 24 h in H_2_-saturated buffer solution (50 mM Tris–HCl, pH 8.0) that contained 0.1 mM NAD^+^, 20 mM **6**, 0.4 mg mL^−1^ ADH-105, and 1 vol% DMSO (see S1.5.4 for more detail). Entries 1 and 3–4: 0.5 mL reaction volume mixed under a flow of H_2_ by shaking at 500 rpm. Entry 2 : 1 mL reaction volume mixed in a pressure vessel that was oscillated at 26 rpm. A relative standard deviation of 9% was determined for Pd TOF (at 2 h) and 1% for all values calculated at 24 h (conversion, TN, TTNs) when entry 2 was performed in triplicate.

b Calculated after 24 h of reacting.

c Mol **7** per mol Pd per h, calculated after 2 h of reacting.

d 0.05 mM NAD^+^ was used.

e Entry 4: shaken under typical conditions for 3 h, then any **6** and **7** were extracted from the reaction by addition and removal of a heptane layer, then a fresh aliquot of **6** was added such that [**6**] = 20 mM and DMSO = 1 vol%.

High NAD^+^ turnover numbers (TN, mol **7** per mol NAD^+^) up to 384 (entry 3) indicated that the Pd did not appreciably decompose the cofactor. This improves upon existing chemo-bio NAD(P)^+^ TN by >7-fold,^18,19,22,43–46^ and further modifications in substrate concentration may provide turnover numbers suitable for commercial application (>1000).^47^ The NAD^+^ reductase total turnover numbers (TTN, mol **7** per mol NAD^+^ reductase) up to 14 000 indicated that the immobilised enzyme was stable to the reaction conditions. High TTN of Pd (up to 2,960, entry 3) suggests it was not significantly impacted by cofactor poisoning nor enzyme inhibition, both of which are common compatibility issues in homogeneous chemo-bio cofactor recycling.^20,38^ High cofactor and Pd turnovers (220 and 3,390, respectively) were also achieved during a reaction in which the organic components were extracted into heptane after 3 h, then fresh **6** in DMSO was added to the reaction and allowed to mix for another 21 h (entry 4).

4′-Chloroacetophenone (**8**) can undergo Pd-catalysed hydrodehalogenation to form **6** or **7** (entry 1, Table 3) or can be reduced by ADH-105 to give **(S)-9** (entries 2–5, Table 4; see Fig. S15† for GC-FID spectra). Under conditions described in S1.5.4 (using 5 mM **8**), the chemo-bio/C catalyst had poor chemoselectivity when 4 bar H_2_ was used (entry 2, Table 4), but the chemo/C+bio/C catalyst provided **(S)-9**, exclusively (>99% ee, entry 3). Under ambient conditions and increased chemo-bio catalyst loading (see Table S7† for optimisation), chemo-bio/C and chemo/C+bio/C catalysts each gave **(S)-9** as the only product in 81% and 91% conversions, respectively (>99% ee, entries 4–5). This method is therefore tolerant of labile groups when used under ambient conditions, circumventing the chemo- and enantioselectivity issues encountered when unmodified Pd/C was used.

**Table 4:** Chemo-bio catalysed reduction of **8**^a^

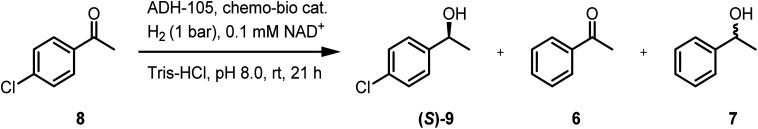					
Entry	Reaction volume (mL)	*P*_H_2__	Catalyst type, loading (mg mL^−1^)	Conversion of **8** (%)	Product ratio **9** : **6** : **7**
1	1	1 bar	1 wt% Pd/C, 0.1	13	0 : 1 : 0
2	1	4 bar	Chemo-bio/C, 0.4	31	37 : 5 : 1
3	1	4 bar	Chemo/C+bio/C, 0.4	14	1 : 0 : 0
4	0.5	1 bar	Chemo-bio/C, 0.8	81	1 : 0 : 0
5	0.5	1 bar	Chemo/C+bio/C, 0.8	91	1 : 0 : 0

a Reaction followed S1.5.4 using 0.1 mM NAD^+^, 5 mM **8**, and 0.4 mg mL^−1^ ADH-105. Reactions performed at 4 bar were placed in a pressure vessel that was charged to 4 bar H_2_ then oscillated at 30 rpm. Reactions performed at 1 bar were placed in a shaker plate under a steady stream of H_2_ and shaken at 500 rpm.

## 3. Conclusions

The high metal and enzyme TOFs and TTNs achieved in this work highlight the use of carbon supports as a general methodology for designing dual-active chemo-bio catalysts. Demonstrated here for NAD^+^ recycling, each component is simultaneously active and selective for its desired reaction. We show that the activity of metal and enzyme catalysts can be tuned by varying the ratio of catalysts for two half reactions, with non-desirable metal activity prevented at low metal/high enzyme loadings and mild conditions. Highly selective hydrogenation reactions were achieved with low waste production relative to product mass (*E* factor: 2.4–3.0, see eqn S1 in ESI† for details).^48^ The methods demonstrated here have the potential to be expanded for combining other metal and enzyme reaction steps in synthetically useful ways.

## Author contributions

X. Z., S. E. C., C. Z., N. G., H. A. R., and K. A. V. all helped to design experiments. X. Z., S. E. C., and C. Z. conducted experiments. X. Z., S. E. C., and H. A. R. analysed data relating to catalyst activity and selectivity. C. Z. and N. G. analysed data relating to catalyst characterisation. All authors discussed the results and commented on the manuscript. N. G., H. A. R., and K. A. V. supervised the project.

## Conflicts of interest

There are no conflicts to declare.

## Supplementary Material

SC-012-D1SC00295C-s001
