# Supplementary material for: Chemo-bio catalysis using carbon supports: application in H2-driven cofactor recycling
Source: Chem Sci. 2021 May 7;12(23):8105–14. doi: 10.1039/d1sc00295c (PMC8208311; doi:10.1039/d1sc00295c)
Supplement: SC-012-D1SC00295C-s001 [file SC-012-D1SC00295C-s001.pdf]

Electronic Supplementary Material (ESI) for Chemical Science.
This journal is © The Royal Society of Chemistry 2021

Supplementary Information for

Chemo-bio catalysis using carbon supports: application in H_2-driven cofactor recycling

X. Zhao,[1] S. E. Cleary,[1] C. Zor,[2] N. Grobert,[2]* H. A. Reeve,[1]* and K. A. Vincent[1]*

[1]Department of Chemistry, University of Oxford, Inorganic Chemistry Laboratory, South Parks Road, Oxford, OX1 3QR.

[2]Department of Materials, University of Oxford, Parks Road, Oxford, OX1 3PH.

Correspondence to: nicole.grobert@materials.ox.ac.uk, holly.reeve@chem.ox.ac.uk, kylie.vincent@chem.ox.ac.uk

Table of Contents

S1. Supplementary Materials and Methods S2

S2. Supplementary Results and Discussion S8

S3. References S28

S1. Supplementary Materials and Methods

S1.1. General reagents

Buffer salts (Sigma Aldrich), NAD^+ and NADH (Prozomix), Rh/C (5 wt%, Alfa Aesar), Ir/C (1 wt%, Alfa Aesar), Ru/C (5 wt%, Alfa Aesar), Pt/C (5 wt%, 1 wt%, Alfa Aesar), Pd/C (10 wt%, Sigma Aldrich; 3 wt%, 1 wt%, Alfa Aesar), carbon black particles (Black Pearls 2000 "BP2000", Cabot Corporation), ketone substrates (**6** and **8**) and GC standards (***(S)*-7**, ***rac*-7** and ***(S)*-9**) were all used as received. Pt/C (20 and 60 wt% Alfa Aesar) and Pd/C (0.5 wt%, Alfa Aesar) were obtained as large granules (mm-cm particle size) and were mechanically ground using a mortar and pestle such that the resulting fine particles could be easily handled by a 1-100 µL pipette as a slurry. All commercial catalyst carbon material types are listed in Table S1. All solutions were prepared with MilliQ water (Millipore, 18 MΩcm).

Table S1. Carbon material type of commercial chemo/C catalysts

Entry	Chemo/C catalyst	Carbon type
1	60 wt% Pt/C	Carbon black
2	20 wt% Pt/C	Carbon black
3	5 wt% Pt/C	Activated carbon
4	1 wt% Pt/C	Activated carbon[a]
5	10 wt% Pd/C	Activated carbon
6	3 wt% Pd/C	Activated carbon
7	1 wt% Pd/C	Activated carbon
8	0.5 wt% Pd/C	Activated carbon[b]
9	5 wt% Rh/C	Activated carbon
10	5 wt% Ru/C	Activated carbon
11	1 wt% Ir/C	Activated carbon
12	BP2000	Carbon black

[a]Paste form. [b]Granular form.

S1.2. Enzymes

NAD^+ reductase (I64A variant of the NAD^+ reducing soluble hydrogenase from *Ralstonia eutropha*, in which the inherent hydrogenase moiety is inactivated, molecular weight 170 kDa)[1,2] was isolated and purified following published protocols,[3] then stored at −80 °C as flash frozen aliquots in 5% glycerol Tris-HCl (100 mM, pH 8 at room temperature). Alcohol dehydrogenase (ADH-105) was generously provided by Johnson Matthey in the His-tagged and purified form and stored as a powder at −20 °C.

S1.3. Chemo-bio catalyst preparations

Co-immobilised "chemo-bio/C" catalyst for NAD^+ reduction. On the benchtop in a round bottom flask under an atmosphere of N_2 (balloon or steady bubbling), 20 mg/mL of Pd/C particles (0.5–3 wt%) were dispersed by stirring in Tris-HCl buffer (50 mM, pH 8.0). The specified amount of NAD^+ reductase was added to the particle slurry and left to stand in the round bottom flask over ice for 60 min. The catalyst was then divided into necessary quantities for experiments, and subjected to NAD^+ reducing conditions.

Mixed carbon agglomerate "chemo/C+bio/C" catalyst used for NAD^+ reduction and ketone reductions. In a glovebox (Glove Box Technology) under a protective N_2 atmosphere (O_2 < 1 ppm), BP2000 and 1 wt% Pd/C were separately suspended to 20 mg/mL in deoxygenated Tris-HCl buffer (50 mM, pH 8.0, 22 °C), dispersed using a 100–1000 µL and a 10–100 µL pipette, respectively, then sonicated 2 × 30 min. Between rounds of sonication, a 10–100µL pipette was used to disperse and mechanically break up particles. Carbon and Pd/C slurries were allowed to cool in an ice block. The NAD^+ reductase was slowly thawed over ice inside of the glovebox. The designated amount of NAD^+ reductase and BP2000 were combined in a centrifuge tube, mixed using a pipette, and allowed to incubate at 4 °C for 60 min. The designated volume of Pd/C slurry was then added to the enzyme on carbon slurry, mixed well, separated into aliquots if necessary, and then used for NAD^+ reduction or ketone reduction by cofactor recycling.

Co-immobilised "chemo-bio/C" catalyst for ketone reduction using cofactor recycling. In a glovebox (Glove Box Technology) under a protective N_2 atmosphere (O_2 < 1 ppm), Pd/C (0.5 or 1 wt%) was suspended in deoxygenated Tris-HCl buffer (50 mM, pH 8.0, 22 °C), dispersed using a 10–100 µL pipette, then sonicated 2 × 30 min. Slurry was allowed to cool in an ice block, then the designated amount of NAD^+ reductase and Pd/C were combined in a centrifuge tube, mixed using a pipette, and allowed to incubate at 4 °C for 60 min. Chemo-bio catalyst could then be divided into smaller aliquots or used for ketone reduction without further modification.

S1.4. Carbon support characterisation

Commercially purchased BP2000 and 1 wt% Pd/C were characterised without further modification. The 0.5 wt% Pd/C was ground to a fine powder as described in S1.1. The {BP2000 + Pd/C (1 wt%)} carbons were mixed by pipetting the two unmodified carbon types as a slurry, which was then dried at 70 °C under vacuum. Carbon supports were tested by a range of methods: scanning electron microscopy (SEM), energy-dispersive X-ray spectroscopy (EDX), Raman spectroscopy, X-ray powder diffraction analysis (XRD) and nitrogen adsorption measurements, the results from which are summarised in S2.4. SEM images and EDX spectra were recorded using a Zeiss Merlin FE-SEM instrument. SEM images were taken with an Inlens secondary electron detector under 5 kV EHT and EDX analysis was done with an Oxford instruments Xmax 150 EDX. Raman spectra were obtained with a JY Horiba Labram Aramis imaging confocal Raman microscope equipped with a green laser (532 nm excitation). XRD analyses were performed using a Rigaku MiniFlex 600 X-ray diffractometer employing CuK_α radiation (operated at 15 mA, 40 kV). Micromeritics Gemini VII BET (Brunauer-Emmett-Teller) surface area analyser was used to investigate the specific surface area, pore size and pore volume of the samples by nitrogen adsorption measurements. Prior to nitrogen adsorption at 77 K, the samples were dried at 160 °C under N_2 overnight.

S1.5. Reaction methods

S1.5.1. Metal on carbon "chemo/C" catalysed NAD$^+$ reduction. An Asynt OCTO mini parallel reactor, in which each reaction tube contained a magnetic stir bar, was flushed under a steady flow H_2. While under H_2, each reaction tube was charged with 2 mL of 5 mM NAD$^+$ in Tris-HCl (50 mM, pH 8.0, room temperature) and stirred for 30 min. Chemo/C catalysts (0.32 mg, treated under a flow of N_2 for 10 min prior to use) were then transferred into their respective reaction tubes using 0.2 mL of the H_2-flushed NAD$^+$ solution, and stirred under a steady flow of H_2 at room temperature. Aliquots were taken for analysis via syringe and needle at 30 min, 2 h, and 17 h. Product compositions were monitored by UV-visible spectroscopy and ^{1}H NMR, and HPLC was used for the last time point to confirm composition against the other methods.

S1.5.2. In situ chemo-bio catalysed NAD$^+$ reductions. Reactions were carried out under H_2 on the benchtop in a UV-visible spectroscopy quartz cuvette (path length 1 cm) that was capped with a rubber septum fitted with two needles to allow gas to flow through the headspace. A volume of 800 µL of H_2-saturated 0.1 mM NAD$^+$ in Tris-HCl (50 mM, pH 8.0, room temperature) were injected into the cuvette. About 0.2 mL of this solution was then used to transfer the catalyst (20 µL slurry, treated under a flow of N_2 for 10 min prior to use) into the cuvette via needle and syringe, and reaction was monitored by in situ UV-visible spectroscopy. Reaction mixture was agitated by inversion (or by magnetic stir bead when specified). Upon reaction conclusion, mixture was centrifuged to remove solid catalyst (10,000 × g, 3 min) and a 100 µL aliquot was taken for HPLC analysis to confirm product composition.

S1.5.3. Ex situ chemo-bio catalysed NAD$^+$ reductions. Reactions were carried out in either an H_2-flushed round bottom flask equipped with a magnetic stir bar or in 1.5 mL centrifuge tubes (Eppendorf) that were punctured with holes in the lid (Ø 1.0 mm) and placed on a container on a shaker plate designed and made in house such that multiple tubes could shake at 500 rpm under a continuous flow of H_2 through an enclosed headspace with a gas inlet and outlet. For the round bottomed flask, 800 µL of the indicated [NAD$^+$] in Tris-HCl (50 mM, pH 8.0, room temperature) was added to the reaction vessel, and 0.2 mL of this solution was used to transfer the catalyst slurry (20 µL, treated under a flow of N_2 for 10 min prior to use) via needle and syringe, and the reaction was left to stir at room temperature under a continuous flow of H_2 (1 bar). For the centrifuge tube, 800 µL of the indicated [NAD$^+$] in Tris-HCl (50 mM, pH 8.0) was added to the reaction tubes containing the chemo-bio/C catalysts (20 µL slurry), then the tube was placed in the shaker plate to mix under a continuous flow of H_2. Reaction progress was monitored by taking aliquots for analysis using UV-vis spectroscopy (and ^{1}H NMR when specified).

S1.5.4. Chemo-bio catalysed NADH recycling for ketone reduction by ADH-105. In a glovebox (Glove Box Technology) under a protective N_2 atmosphere (O_2 < 0.1 ppm), a solution of NAD^+ was diluted to the designated concentration in deoxygenated Tris-HCl (50 mM, pH 8.0, 22 °C) and saturated with H_2. All reactions were conducted on a 0.5 or 1 mL scale in sealed 1.5 mL centrifuge tubes (Eppendorf) that were punctured with one hole in the lid (Ø 1.0 mm). In sequence, designated volume of H_2-saturated NAD^+ solution was used to transfer the chemo-bio catalyst into the reaction tube, then 0.2 mL of that slurry was used to transfer the ADH-105 into the tube via syringe and needle. The ketone was then added to the reaction mixture as a stock solution in DMSO (diluted such that the total volume of DMSO was 1%). The tube was sealed and placed in the designated reaction vessel. Reaction tubes that were shaken under a flow of H_2 were placed in a container on a shaker plate designed and made in house such that multiple tubes could shake at 500 rpm under a continuous flow of H_2 through an enclosed headspace with a gas inlet and outlet. Reaction tubes that were oscillated under an elevated pressure were placed in a Büchi Tinyclave pressure vessel that was charged to the designated pressure of H_2, then was removed from the glovebox and placed on a Stuart® mini see-saw rocker set to 26 rpm. Reaction progress was monitored at 2 h and 24 h by chiral GC-FID. Final product composition and purity could be confirmed using ^1H NMR.

S1.6. Reaction analysis of NAD^+ reductions

The extent of NAD^+ conversion and analysis of product distribution was calculated by one or more of the following: ^1H NMR spectroscopy, HPLC, and UV-vis spectroscopy. Prior to analysis, the reaction mixture was centrifuged (10,000 rpm, 4 °C, 3 min) to remove solid catalysts and the supernatant was analysed.

S1.6.1. ^1H NMR spectroscopy

NAD^+ reduction product distribution was quantified by ^1H NMR spectroscopy when indicated. Following removal of the solid catalyst (see S1.6), a total volume of 600 µL NMR solution was prepared containing 5 mM DMSO in D_2O in a Norell® SelectSeries™ 5 mm 400 MHz NMR spectroscopy tube. All NMR spectra were obtained from a Bruker Advance III HD nanobay (400 MHz) and parameters used for acquiring NMR spectra are summarised in Table S2. Peaks were referenced against DMSO (δ = 2.72 ppm), where the latter two peaks were referenced against and internal standard (sodium trimethylsilylpropanesulfonate, DSS) (δ = 0.00 ppm). Diagnostic peaks for **1**, **2**, and **3** and representative spectra are displayed in Figure S2 and S3.

Table S2. Parameters used to acquire NAD^+ reduction product composition

**Experiment**	^1H
**RF pulse energy (MHz)**	400.13
**Temperature (K)**	298
**Number of scans**	As required
**Pulse width (µs)**	14
**Spectral width (Hz)**	8000
**Acquisition time (s)**	4.09
**Relaxation delay (s)**	1.00

S1.6.2. UV-visible spectroscopy

UV-visible spectra were recorded by a Cary 60 spectrophotometer with a cell holder (Agilent) with the option for magnetic stirring using a quartz cuvette (path length 1 cm, cell volume 1 mL, Hellma) and 50 mM Tris-HCl (pH 8.0, room temperature) was used as a baseline scan. Particles in solution led to a uniform light scattering across the length of the monitored spectral region (λ = 200–800 nm), which was corrected for during data processing by a simple baseline correction based on the absorbance at λ = 500 nm. For ex situ analysis, the solution containing cofactor was diluted to a known concentration [< 0.1 mM].

S1.6.3. HPLC

Different products of NAD^+ reduction could be separated by HPLC (Shimadzu Prominence) and products whose standard was available could be identified. Aliquots (100 µL) of reaction mixture were taken and centrifuged to remove catalyst particles (10,000 × g, 3 min) for analysis. Products were separated on a SeQuant® ZIC®-HILIC 5 µm, 200 Å column (150 × 4.6 mm) with a guard column (20 × 2.1 mm) using a mobile phase of MilliQ water and HPLC grade acetonitrile buffered with ammonium acetate (20 mM) using method described in Table S3. Two wavelengths (260 nm and 340 nm) were used to detect the elutes. Peaks that eluted at 18.3 min and 19.8 min were identified as 1,4-NADH (**2**) and NAD^+ (**1**) compared to standards, where other peaks shown at 18.7 and 20.8 min were classified into by-products **3-5**.

Table S3. HPLC method used to separate and identify products of NAD^+ reduction

**Column Temperature**	60 °C	
**Flow rate**	1 mL min^{-1}	
**Gradient profile**	Time (minutes)	Acetonitrile (vol %)
	0 – 1	100
	1 – 30	100 – 60
	30 – 35	60 – 40
	35 – 36	40 – 100
	36 – 42	100
**Injection volume**	2 or 10 µL	

S1.7. Reaction analysis of ketone reductions

For reductions of **6** and **8**, the extent of conversion of and enantiomeric excess (% *ee*) of the **(*S*)-7** and **(*S*)-9** products were determined by chiral-phase GC-FID. Aliquots (50 µL) of reaction mixture were taken for analysis at 2 h and 24 h, extracted into 800 µL EtOAc, and centrifuged to separate catalyst particles (12,000 × g, 2 min) if needed. Following partitioning, 600 µL of the EtOAc layer was removed and dried over Na_aSO_4, then taken for GC analysis. See details of results in S2.10–S2.12.

S1.7.1. Chiral-phase GC-FID method

Column: CP-Chirasil-Dex CB (Agilent), 25 m length, 0.25 mm diameter, 0.25 µm (film thickness), fitted with a guard of 10 m undeactivated fused silica of the same diameter
Carrier: He (CP grade), 170 kPa (constant pressure)
Inlet temperature: 200 °C
Injection conditions: Splitless with split flow 60 mL/min, splitless time 0.8 mins, purge 5 mL/min. Injection volume = 0.5 µL.
Detection: FID (H_2 = 35 mL/min, air = 350 mL/min, makeup N_2 = 40 mL/min, temp = 200 ˚C)
Oven heating profile:

Time (minutes)	Temperature
0 – 5	Hold at 70˚C
5 – 30	Ramp to 120 ˚C at 2 ˚C/min
30 – 36	Ramp to 180 ˚C at 10 ˚C/min
36 – 45	Hold at 180 ˚C for 5 minutes

Compound retention times (reduction of **6***)*:

Time (minutes)	Compound
10.18	Acetophenone (**6**)
12.43	(*R*)-1-Phenylethanol (**7**)
12.62	(*S*)-1-Phenylethanol (**7**)

Compound retention times (reduction of **8***)*:

Time (minutes)	Compound
13.05	4’-Chloroacetophenone (**8**)
14.97	(*R*)-4’-Chloro-1-phenylethanol (**9**)
15.13	(*S*)-4’-Chloro-1-phenylethanol (**9**)

S2. Supplementary Results and Discussion

S2.1. Exemplary metal/C-catalysed NAD^+ reduction performed in triplicate to determine the reproducibility of experiments

The experiment from Fig. 2a in which 3 wt% Pd/C is stirred with NAD^+ under H_2 was performed in triplicate to determine the reproducibility of experiments (Figure S1). At 17 h, the product compositions were determined by ^1H NMR spectroscopy. The relative standard deviations for 1,6-NADH (**3**), other/missing (**4** and **5**), and NAD^+ are 5.1, 7.8 and 6.9%, respectively.

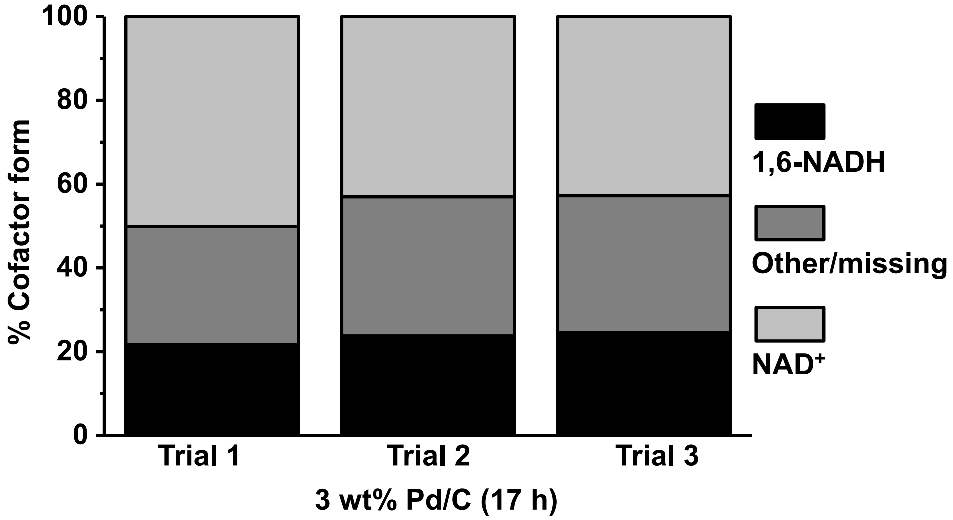

Figure S1. Cofactor compositions from H_2-driven NAD^+ reduction by 3 wt% Pd/C, determined by ^1H NMR spectroscopy at 17 h. Reaction conditions see S1.5.1.

S2.2. Exemplary analytical data to determine product composition for H_2-driven NAD^+ reduction selectivity

Due to the challenge of detecting and quantifying the presence of undesired NAD^+ reduction products, we used three analytical methods for verification. The products vary in their presentation of diagnostic signals across the three analytical techniques. Here we present two case examples that resulted in different levels of 1,4-NADH (**2**) selectivity, and use ^1H NMR spectroscopy, UV-visible spectroscopy, and HPLC to verify product composition. We could identify and quantify the formation of undesired 1,6-NADH (**3**) using ^1H NMR spectroscopy based on the diagnostic singlet (7.27 ppm, grey) that is known to appear downfield from the diagnostic singlet (6.95 ppm, green) that corresponds with **2**.[6] Over-reduced cofactor forms (e.g. **4**) were also assigned to the peaks between 1–2 ppm (saturated hydrocarbons).[7]

Figure S2 shows an NAD^+ reduction that was initially selective for **2** (diagnostic peaks shown in green), however this selectivity was diminished after 17 h. This was first established using ^1H NMR spectroscopy (grey, Figure S2a). A corresponding small shoulder between 290–310 nm appeared by UV-visible spectroscopy. Two new peaks by HPLC further verified the formation of side-products (likely **3** and other inactive cofactor).

When NAD^+ reductions were not selective from the start, we could confidently identify the side-products by ^1H NMR spectroscopy (grey, Figure S3a). This gave confidence that the shoulder appearing between 290–310 nm by UV-visible spectroscopy were due to the formation of side-product, and three impurity peaks were identified by HPLC (Figure S3c).

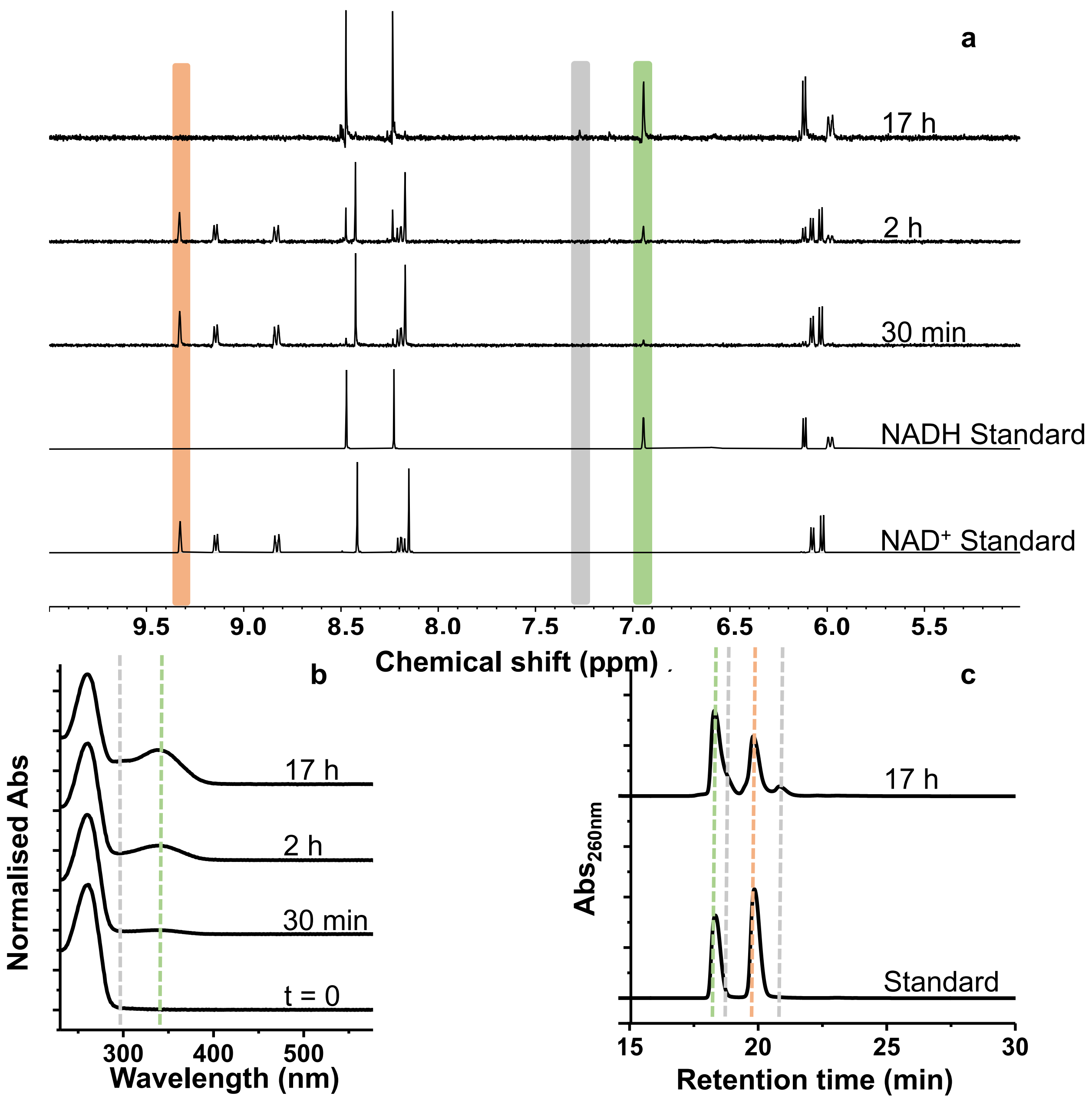

Figure S2. Representative example of analytical data for the determination of cofactor composition of NAD^+ reduction reaction that had some selectivity for 1,4-NADH (catalyst used: 20 wt% Pt/C that corresponds with Fig. 2 in the main text). Reaction conditions see S1.5.1.. a. ^1H NMR spectra of three defined time points and cofactor standards taken in 5:1 Tris-HCl buffer (50 mM, pH 8, room temperature)/D_2O. b. UV-visible spectra of three defined time points. c. HPLC traces detected at 260 nm after 17 h of reaction and standard of mixture of NAD^+ (**1**) and 1,4-NADH (**2**). Orange region: NAD^+. Grey region: inactive form of NADH and by products (**3**–**5**). Green region: 1,4-NADH.

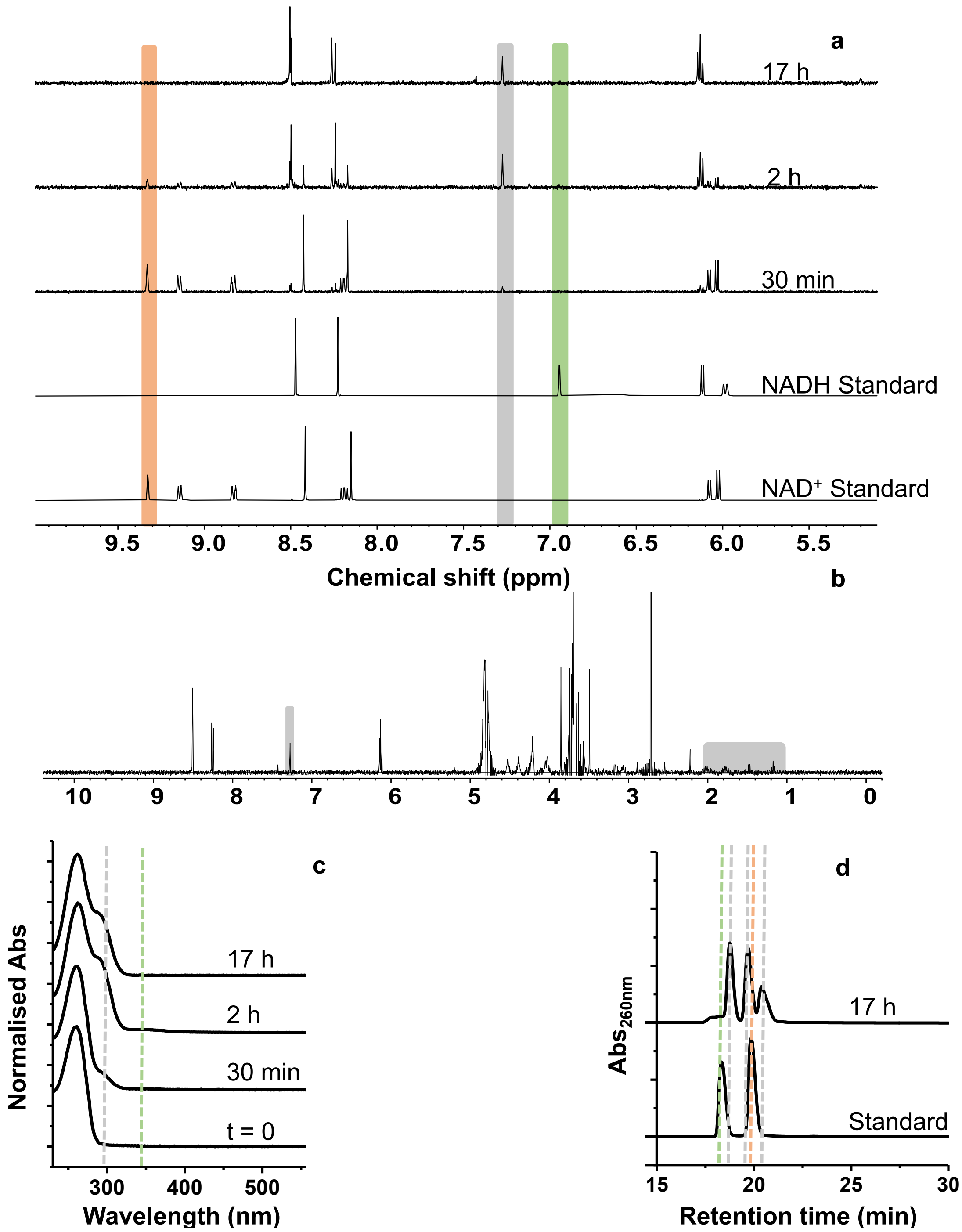

Figure S3. Representative example of analytical data for the determination of cofactor composition of NAD^+ reduction reaction that had no selectivity for 1,4-NADH (catalyst used: 10 wt% Pd/C that corresponds with Fig. 2 in the main text). Reaction conditions see S1.5.1. a. ^{1}H NMR spectra of three defined time points and cofactor standards. b. Full ^{1}H NMR spectra at 17 h includes peaks resulting from saturated hydrocarbons (0–2 ppm) c. UV-visible spectra of three defined time points. d. HPLC traces detected at 260 nm after 17 h of reaction and standard of mixture of NAD^+ (**1**) and 1,4-NADH (**2**). Orange region: **1**. Grey region: inactive form of NADH and by products (**3–5**). Green region: **2**.

S2.3. Effect of stirring chemo/C with 1,4-NADH under H_2 or N_2 atmosphere

The UV-visible spectra in Figure S4 show that stirring metal on carbon particles in a solution of **2** under N_2 leads to a decrease in concentration, and might contribute to the "missing" cofactor. No by-products (**3**–**5**) were detected under these conditions, though it appears that **1** was formed in the presence of some metal on carbon particles. A control experiment in the absence of catalyst (not shown) showed a consistent **2** throughout the reaction period.

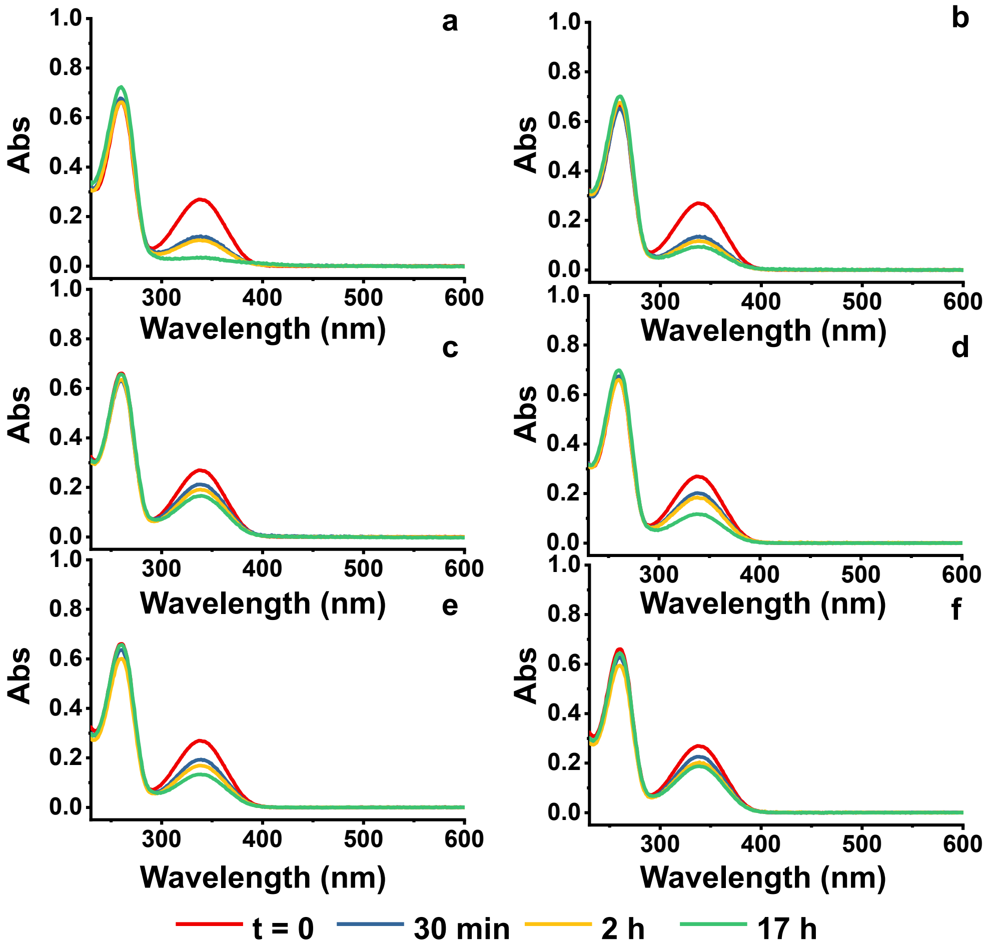

Figure S4. UV-visible spectra of reaction mixtures containing **2** in the presence of different metals on carbon under N_2. Reaction conditions see S1.5.1.with the modification that 5 mM **2** was used instead of NAD^+ (**1**). Catalysts present: a: 60 wt% Pt/C. b: 20 wt% Pt/C. c: 10 wt% Pd/C. d: 3 wt% Pd/C. e: 1 wt% Pd/C. f: 0.5 wt% Pd/C.

Additional control experiments were designed to analyse if **2** was left unchanged under H_2 atmosphere. The ^{1}H NMR results (30 min) and UV-vis spectra (three time points, Figure S5) showing that **2** can convert to by-products **3**–**5** in the presence of chemo/C particles under H_2.

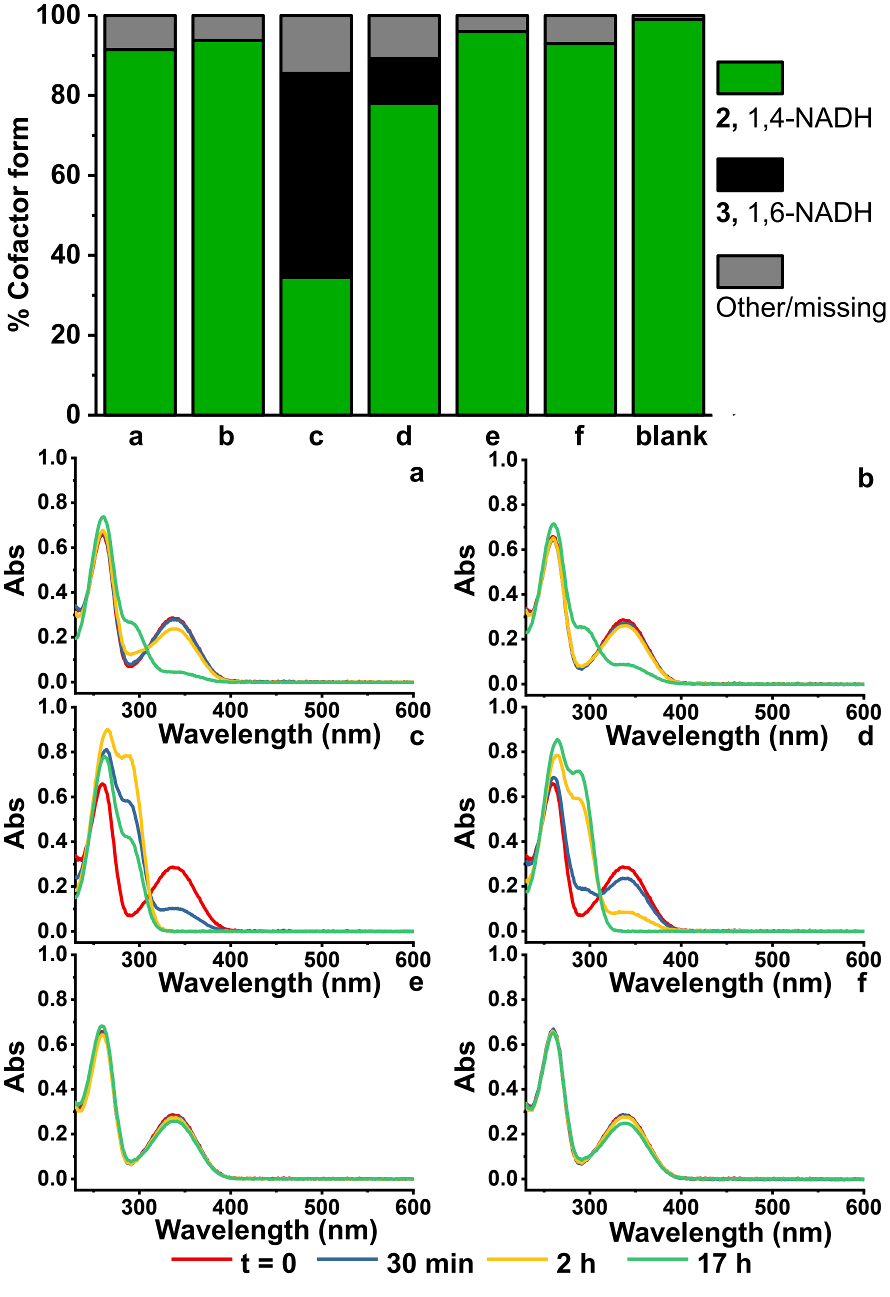

Figure S5. Control experiments to test cofactor composition after stirring **2** in the presence of different metals on carbon under H_2 as analysed by ^{1}H NMR spectroscopy at 30 min (top) and UV-visible spectroscopy at three defined time points (bottom). Reaction conditions: see S1.5.1with the modification that 5 mM **2** was used instead of NAD^+ (**1**). Catalysts used: a: 60 wt% Pt/C. b: 20 wt% Pt/C. c: 10 wt% Pd/C. d: 3 wt% Pd/C. e: 1 wt% Pd/C. f: 0.5 wt% Pd/C.

S2.4. Carbon characterisation results

S2.4.1. X-Ray Diffraction

Diffraction patterns obtained by XRD analysis (Figure S6) show that carbon supports have different crystal structures and that the commercial 0.5 wt%, 1 wt% and 3 wt% Pd-containing supports were prepared by different types of carbon. Presence of Pd can be confirmed by distinct peaks at 2θ of 40.1°, 46.7°, 68.1°, 82.1°, and 86.7° (JCPDS card, file no. 46–1043). Peaks and/or broad profiles present in all carbon samples at 2θ ~25° and 44° are due to graphitic crystallites and correspond to (002) and (100) planes, respectively. The peaks that are not due to carbon and Pd arise from SiO_2 impurity in commercial samples. The X-ray photon scattering intensity increases with decreasing 2θ which is observed in many amorphous materials. Sharper and narrow peaks at 2θ ~25° peaks indicate more ordered graphitic structure, and broad peaks indicate amorphous nature of the materials. Thus the diffraction patterns show that samples are largely amorphous with a low degree of crystallinity.

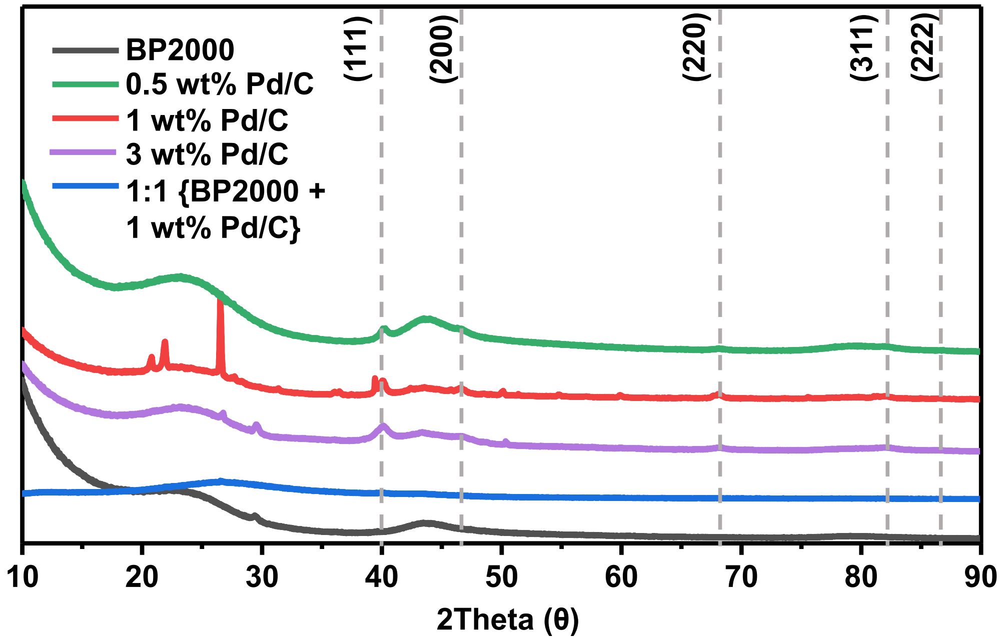

Figure S6. XRD spectra of BP2000 (black), Pd/C (0.5 wt%, green; 1 wt%, red; 3 wt%, purple), and 1 : 1{BP2000 + 1 wt% Pd/C} (blue). Peaks that arise from the presence of palladium are indicated with dashed grey lines.

S2.4.2. Raman spectroscopy

Raman spectroscopy was also used to verify degree of graphitisation of the commercial carbon supports mainly by determining the ratio of peak intensities of the D band (around 1340 cm^{-1}) and G band (around 1575 cm^{-1}) (I_D/I_G), as well as the presence or absence of 2D peak (also referred to as G', around 2675 cm^{-1}, Figure S7). The D band correlates with degree of sp^3 hybridisation and the G band correlates with degree of sp^2 hybridisation (graphitic network), therefore when $I_D/I_G < 1$ the materials are considered to exhibit a higher composition of extended graphitic structure. A 2D peak arises from interactions between any graphene-like planes and contributes to the graphitic content of the carbon.

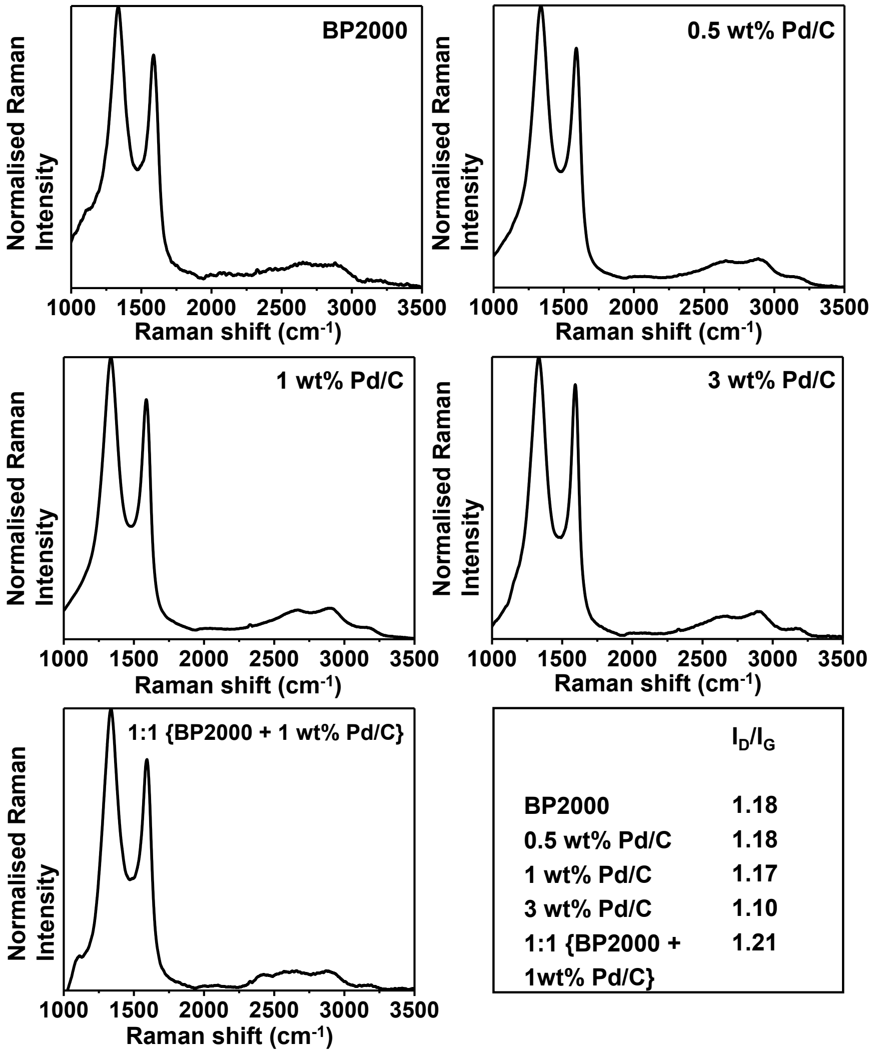

	I_D/I_G
BP2000	1.18
0.5 wt% Pd/C	1.18
1 wt% Pd/C	1.17
3 wt% Pd/C	1.10
1:1 {BP2000 + 1wt% Pd/C}	1.21

Figure S7. Raman spectra of commercial carbon samples showing the degree of graphitisation by I_D/I_G ratio analysis.

The ratio of I_D/I_G was consistently slightly higher than 1 in each sample, which is indicative of the relative amorphous structures of the carbon supports (Figure S7). All carbon samples have broad and weak peaks in the second-order region (2200-3400 cm^{-1}), and BP2000 appears to have a less pronounced wide and single second-order peak which indicates that it is highly disordered.

S2.4.3. BET

The specific surface area, pore diameter and pore volume of the commercial carbon supports were evaluated from the sorption data. Surface areas according to the BET and Langmuir models are reported together. Langmuir model is typically used for microporous materials. The pore characteristics of the carbon supports were studied using the Barrett-Joyner-Halenda (BJH) model (Table S4). Sorption isotherms (Figure S8) indicate that BP2000 has mesopores as well as micropores and display an isotherm between types II and IV with H3 hysteresis. 0.5 wt% Pd/C is microporous and a plateau at high relative pressures indicate type I isotherm. 1 wt% Pd/C and 3 wt% Pd/C display type IV isotherm with H3 hysteresis. They are mainly mesoporous materials but have micropores as well.

Table S4. Surface area and pore characteristics of the carbon supports

Entry	Material	Specific Surface area ($m^2\ g^{-1}$)[a]	Pore Diameter[b] (nm)	Pore Volume[c] ($cm^3\ g^{-1}$)
1	BP2000	1272 ± 23<br>**1949 ± 25**	5.6	1.3
2	0.5 wt% Pd/C	838 ± 23<br>**1241± 1.5**	2.5	0.45
3	1 wt% Pd/C	828 ± 16<br>**1254 ± 13**	4.6	0.69
4	3 wt% Pd/C	768 ± 15<br>**1162 ± 13**	4.1	0.59
5[b]	1:1 {BP2000 + 1 wt% Pd/C}	938 ± 17<br>**1433 ± 18**	6.5	1.1

[a]Models used for specific surface area are BET and Langmuir (bolded). [b]Average pore diameter estimated by BJH desorption (4V/A). [c]Single point measurement at relative pressure 0.95 for pores less than 40 nm.

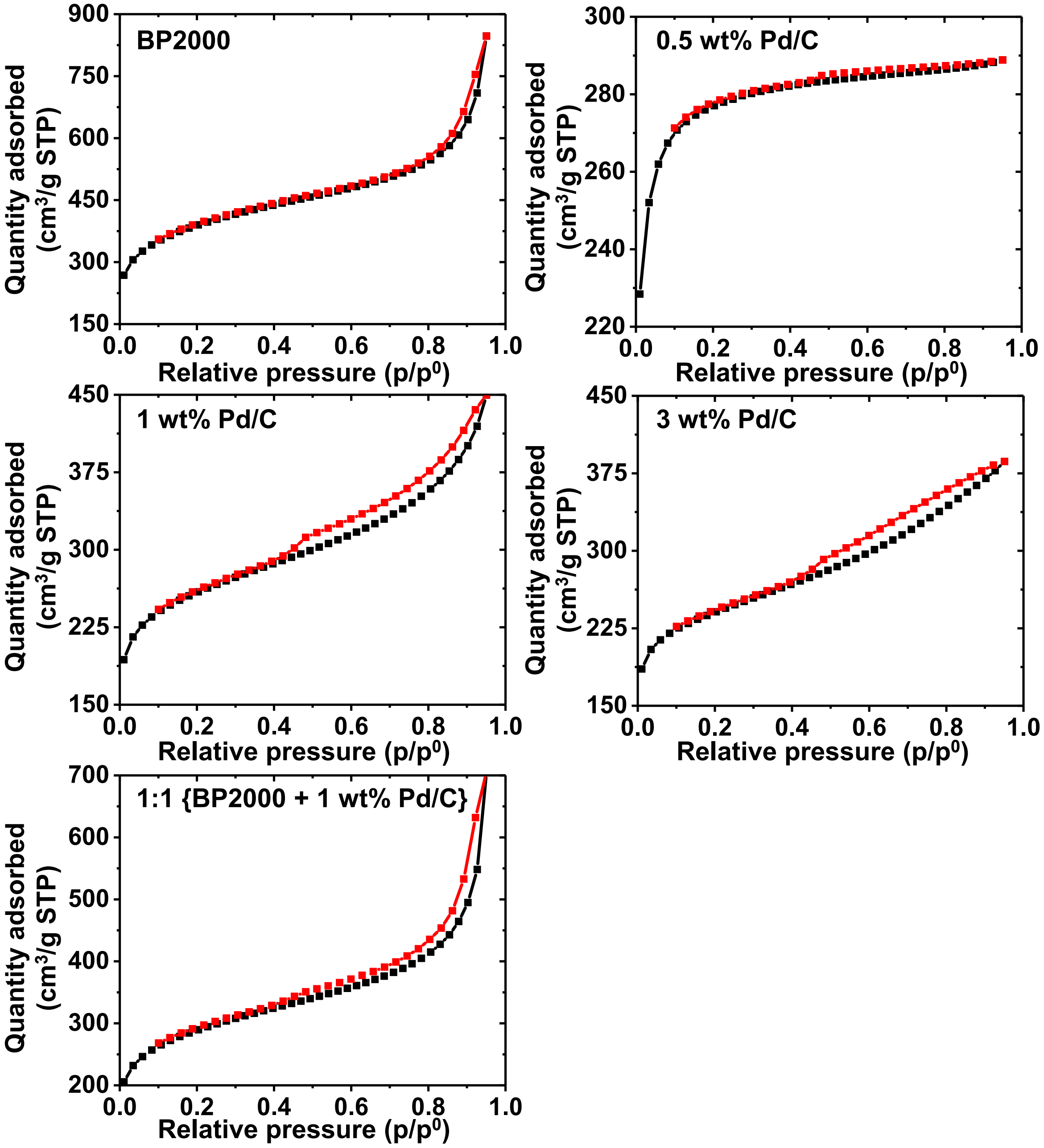

Figure S8. Nitrogen adsorption (black) and desorption isotherms at 77 K for the investigated carbon materials**.**

S2.4.4. Scanning electron microscopy (SEM) and energy dispersive X-ray spectroscopy (EDX)

SEM/EDX was used to study the morphology and determine the elemental surface composition of the commercial carbon supports. SEM micrographs (see Fig. 3 and Figure S9) show that BP2000 has a uniform particle shape distribution. Other commercial supports have small particles and larger agglomerates. The micrograph of the 1:1 mass ratio mixture of BP2000 + 1 wt% Pd/C show the Pd/C support is mostly covered by BP2000 particles. According to the EDX data, in addition to the expected elements (carbon, oxygen, and palladium), a number of other elements were detected which we attribute to steps in the production process (Table S5). The amount of Pd on the surface is greater than that of the bulk, as anticipated. Importantly, the surface palladium of the 0.5 wt% Pd/C (1.5%, entry 3) and the 1:1 mixture of BP2000 + 1 wt% Pd/C (1.4%, entry 4) were comparable. This validates our comparison of the chemo-bio/C catalyst composed of 0.5 wt% Pd/C with the chemo/C+bio/C catalyst composed of 1:1 BP2000 + 1 wt% Pd/C in terms of activity, which relies on Pd NPs on the surface for H_2 oxidation.

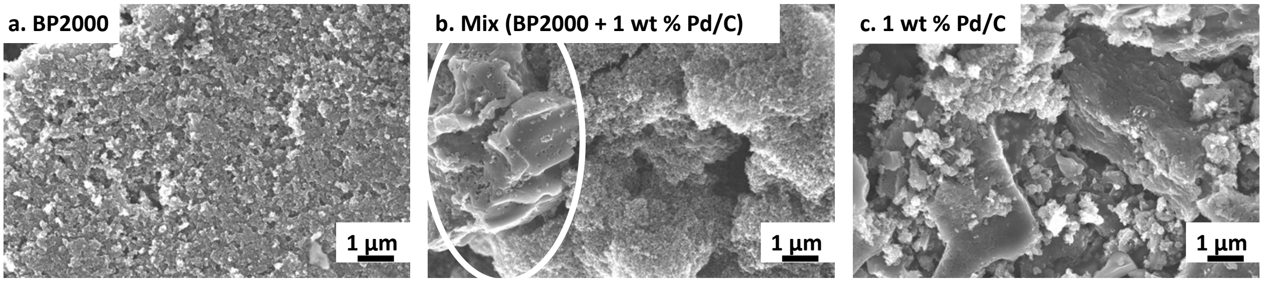

Figure S9. SEM micrographs showing commercial carbon materials and the mixed carbon agglomerate. **a.** BP2000 has more uniform, spherical morphology. **b**. BP2000 and 1 wt% Pd/C mixed as a slurry; circled portion shows Pd/C platelets that were not covered by smaller, spherical BP2000 particles. **c**. 1 wt% Pd/C shows small particles and large agglomerates and platelets.

Table S5. Elemental composition of commercial carbon samples determined from EDX spectroscopy.

Entry	Sample	C (%)	O (%)	Pd (%)	Other elements	% Protein not adsorbed[a]
1	BP2000	93.45	4.2	n.d.[b]	Si (1.8%), S (0.5%), Na (0.05%)	3
2	3 wt% Pd/C	82.53	4.87	11.07	Ca (0.83%), Mg (0.27%), S (0.23%), Na (0.07%), Al (0.13%)	72
3	1 wt% Pd/C	92.1	4.6	2.5	Si (0.45%), S (0.2%), Al (0.15%)	60
4	0.5 wt% Pd/C	95.27	2.8	1.5	Si (0.18%), Na (0.12%), Mg (0.07%), K (0.06%), F (0.02%)	75
5	1:1 {BP2000 + 1% Pd/C}	92.5	4.2	1.4	S (0.6%), Ca (0.5%), Si (0.4%), F (0.3%)	22

[a]200 µg NAD^+ reductase was incubated with 200 µg Pd/C (or 100 µg BP2000 + 100 µg Pd/C for entry 5), then the resulting supernatant was analysed for leftover protein relative to starting protein concentration using a Bradford assay. [b]Not detected.

S2.5. Background activity of NAD^+ reductase for reduction of 1

The background activity of NAD^+ reductase in solution (blue) and immobilised on BP2000 (green) under H_2 was tested following S1.5.2 (Figure S10). Only trace **2** was detected when NAD^+ reductase was used in solution. For reference, results from NAD^+ reduction experiments using co-immobilised chemo-bio/C catalysts are included to show activity when 0.5 wt% Pd is present (black and red). Conversion to **2** was calculated using UV-visible spectroscopy based on the appearance of a peak at 340 nm (ε_{340} = 6.22 mM cm^{-1}).

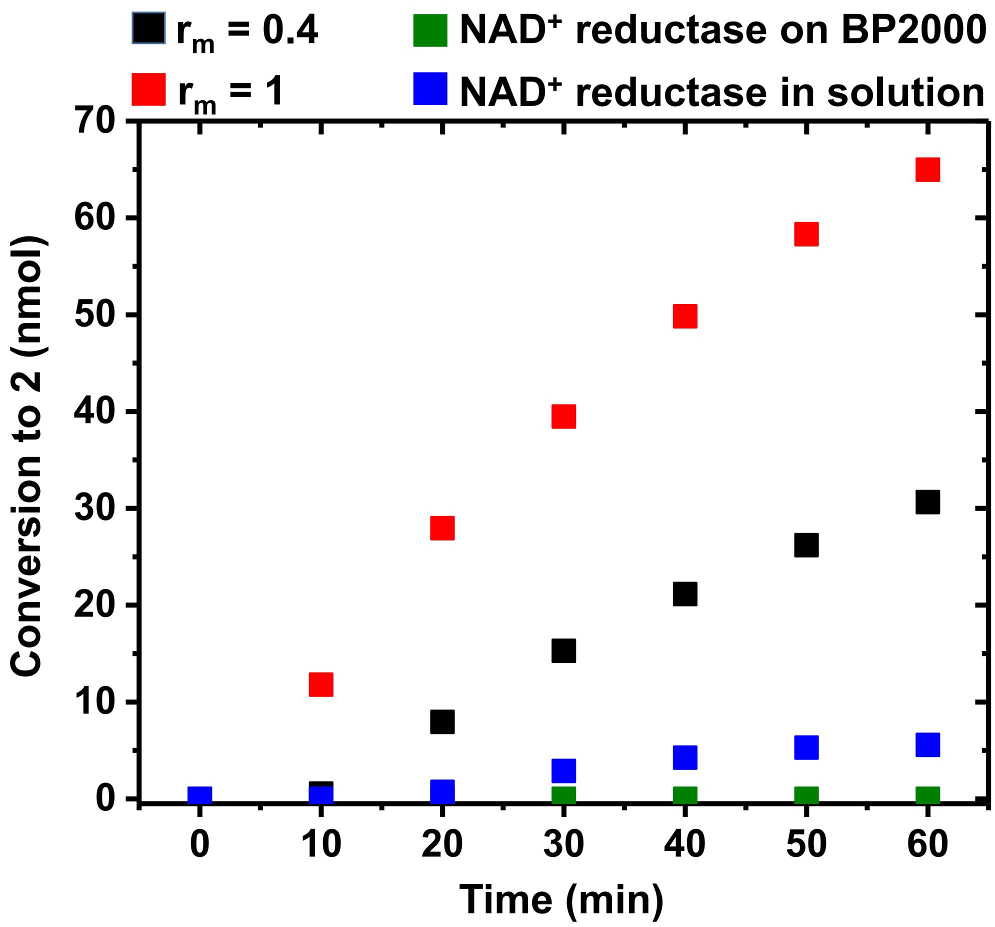

Figure S10. Time course for conversion of NAD^+ (**1**) to 1,4-NADH (**2**) using 40 µg NAD^+ reductase immobilised on 20 µg BP2000 (green), 8 µg NAD^+ reductase in solution (blue), or chemo-bio/C catalysts with varying r_m in which mass of enzyme is increased to correspond with 20 µg 0.5 wt% Pd/C. Reactions conditions see S1.5.2 **(the experiment in green was carried out inside of a glovebox and the reaction was mixed using a magnetic bead).** Conversion was monitored using UV-visible spectroscopy.

S2.6. Control experiment to test NAD$^+$ reductase adsorption onto carbon supports

Bradford assays were performed to determine the concentration of protein in the resulting supernatant after NAD$^+$ reductase and carbon materials were incubated together over 1 h inside of a glovebox on an ice block.

Table S6. Remaining NAD$^+$ reductase after adsorption on 0.5 wt% Pd on carbon to probe reasoning for inferior enzyme activity at increasing r_m.

Entry	r_m	% Protein left in supernatant
1	0.05	1
2	0.1	2
3	0.2	3
4	0.4	7
5	1	15
6	2	40

Catalyst composition: 100 µg of Pd/C with varied mass of NAD$^+$ reductase.

S2.7. Chemo-bio/C catalyst inhibition by elevated [NAD$^+$]

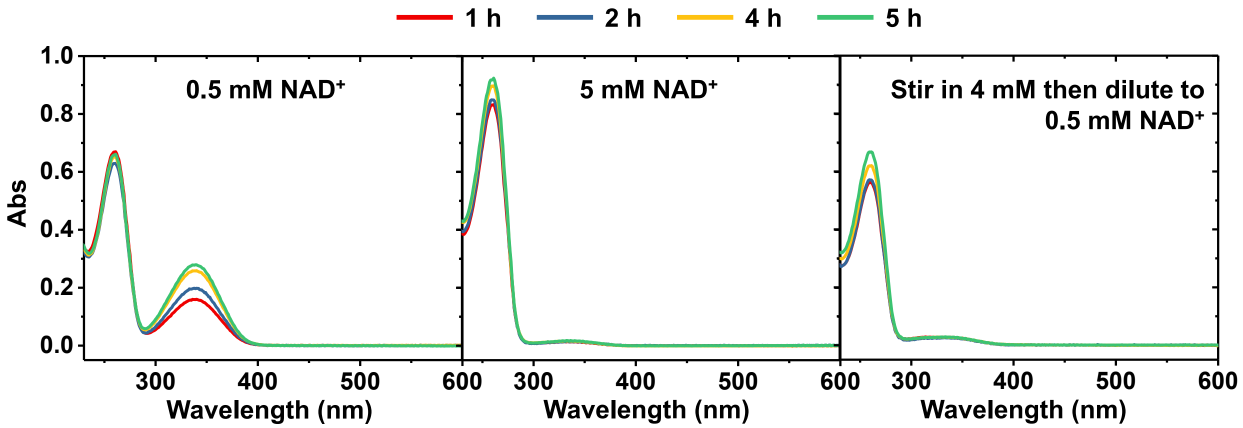

Figure S11. Ex situ UV-visible spectra taken at 1, 2, 4, and 5 h to calculate conversion of NAD$^+$ (**1**) to 1,4-NADH (**2**) using co-immobilised chemo-bio/C catalysts with varying concentration of **1**. Time point sample aliquots taken for analysis were each diluted to 0.05 mM using Tris-HCl prior to scan. Reaction conditions see S1.5.3.using chemo-bio/C catalyst (20 µg 0.5 wt% Pd/C and 40 µg NAD$^+$ reductase, $r_m = 2$).

S2.8. Product distribution from NAD^+ reduction by chemo/C+bio/C

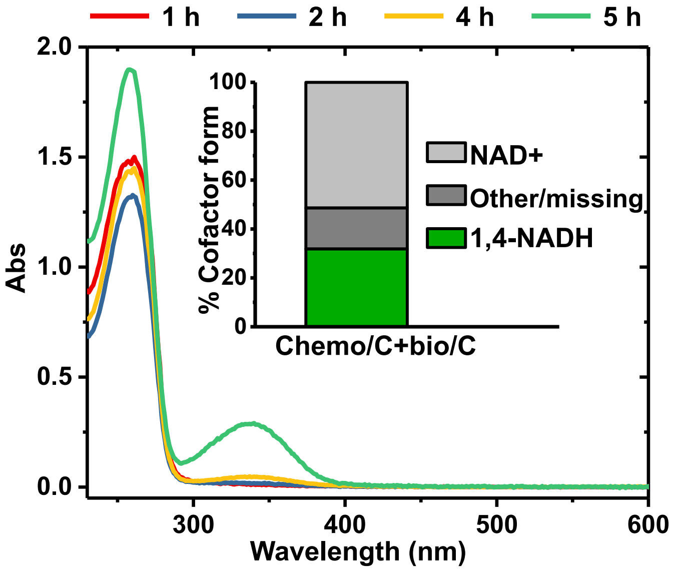

Figure S12. Time course of 1 mM NAD^+ reduction by 20 µg chemo/C+bio/C catalyst (r_m = 0.1) analysed by ex situ UV-visible spectroscopy. Inset: Cofactor compositions after 23.5 h determined by ^1H NMR spectroscopy. Reaction conditions see S1.5.3. Catalyst composition: 10 µg 1 wt% Pd/C, 10 µg BP2000, 2 µg NAD^+ reductase. Peak at 260 nm likely increased due to the evaporation of the reaction solution, increasing [NAD^+].

S2.9. Different preparation of mixed chemo/C+bio/C catalysts and comparison of their activities

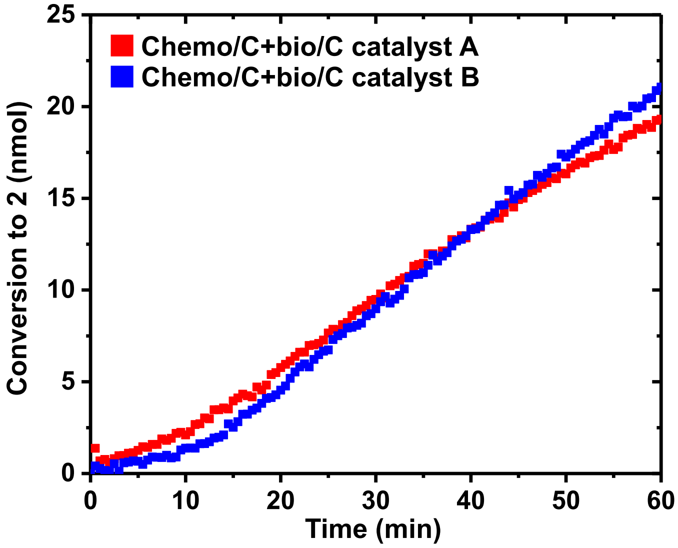

Figure S13. Time course of 0.1 mM NAD^+ reduction by 20 µg chemo/C+bio/C catalyst (r_m = 1) analysed by in situ UV-visible spectroscopy. Reaction conditions see S1.5.2with the modification that experiments were carried out inside of a glovebox and the reaction was mixed using a magnetic bead. Chemo/C+bio/C catalyst A (red): prepared from 20 µg NAD^+ reductase immobilised on 10 µg BP2000, and this was then mixed with 10 µg 1 wt% Pd/C. **Chemo/C+bio/C** catalyst B (blue): prepared by mixing 10 µg BP2000 and 10 µg 1 wt% Pd/C, then 20 µg NAD^+ reductase was immobilised on the mixture of supports.

S2.10. Screening of NADH recycling for the reduction of 8 by ADH-105

Initial experiments that tested the reduction of **8** were based on the optimised conditions used for the reduction of **6** (listed in entries 8 and 10, Table 2). Co-immobilised and mixed carbon agglomerate chemo-bio catalysts were initially subjected to S1.5.4 wherein [**8**] was 20 mM (not tabulated). After 24 h, conversion of **8** was > 5% for both chemo-bio/C and chemo/C+bio/C catalysts, therefore [**8**] was adjusted to 5 mM (results summarised in Table 7). The chemo-bio/C catalyst was not selective for **(*S*)-9** at elevated pressure of H_2, and gave dehalogenated products **6** and **7** in addition to the expected product (entry 1). When used in the shaker plate under a steady flow (1 bar) of H_2, both catalysts were selective for **(*S*)-9** (entries 3-4). Both chemo-bio catalysts afforded **(*S*)-9** in good conversions when a catalyst loading of 0.8 mg/mL was used (entries 5–6).

Table S7. H_2-Driven chemo-bio catalysed reduction of **8**

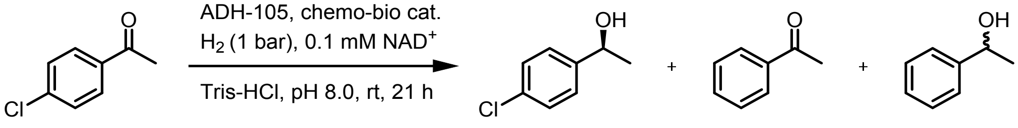

8 **(*S*)-9** **6** **7**

Entry	Reaction Volume (mL)	P_{H_2}	Catalyst type, loading (mg/mL)	Conversion of 8 (%)[a]	Product Ratio 9 : 6 : 7[a]
1	1	4 bar	chemo-bio/C 0.4	31	37 : 5 : 1
2	1	4 bar	chemo/C+bio/C 0.4	14	1 : 0 : 0
3	0.5	1 bar	chemo-bio/C 0.4	70	1 : 0 : 0
4	0.5	1 bar	chemo/C+bio/C 0.4	59	1 : 0 : 0
5	0.5	1 bar	chemo-bio/C 0.8	81	1 : 0 : 0
6	0.5	1 bar	chemo/C+bio/C 0.8	91	1 : 0 : 0
7	1	1 bar	1 wt% Pd/C 0.1	13	0 : 1 : 0

Reaction conditions see S1.5.4 using 0.1 mM NAD^+, 5 mM **8**, and 0.4 mg/mL ADH-105. Reactions performed at 4 bar (entries 1-2) were placed in a pressure vessel that was charged to 4 bar H_2 then left to rock at 30 rpm. Reactions performed at 1 bar were placed in a shaker plate that was left to shake at 500 rpm under a steady flow of H_2 through the headspace of a sealed compartment. [a]Calculated based on chiral GC-FID.

S2.11. Examples of chiral-phase GC-FID spectra from ketone reductions

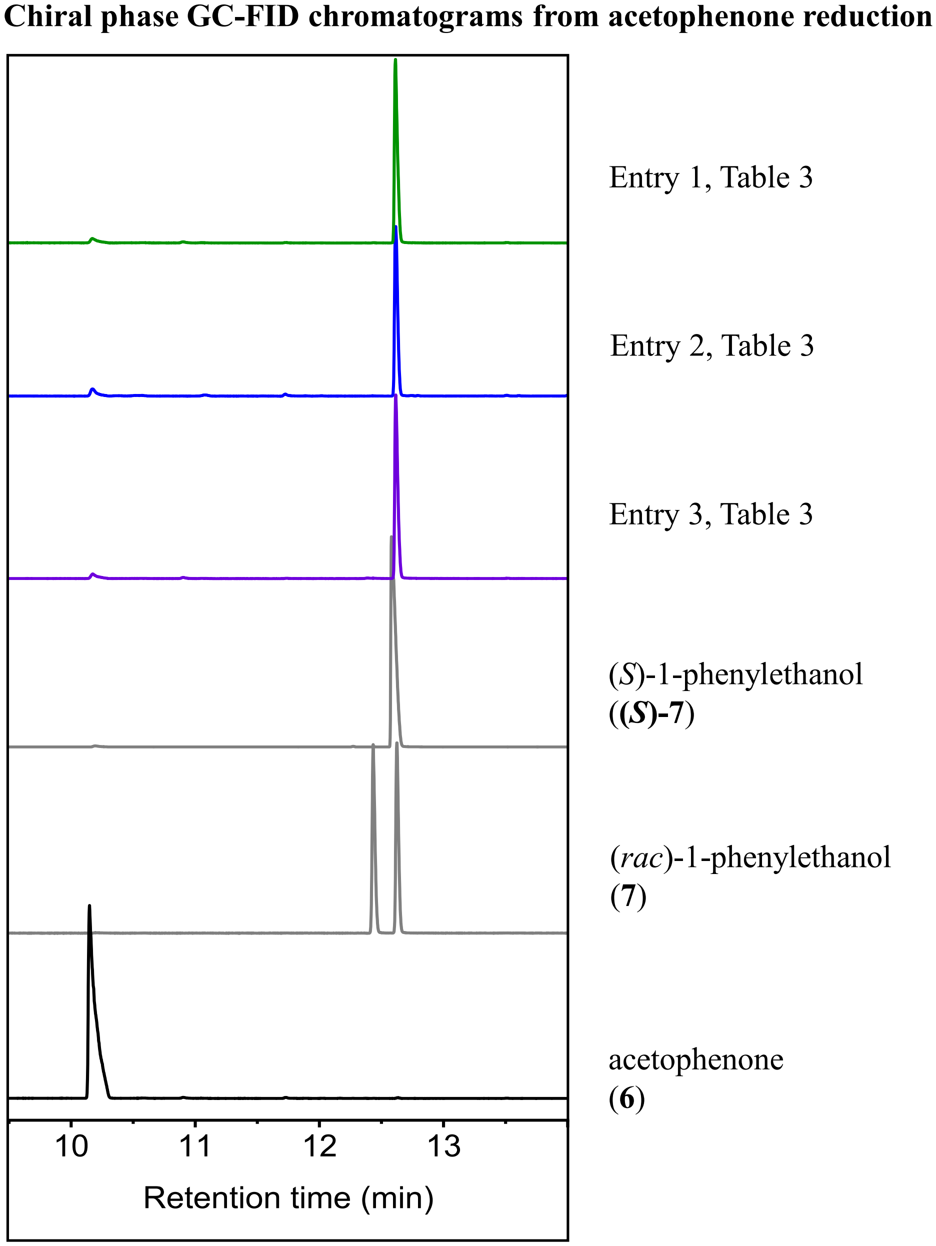

Figure S14. Examples of chiral-phase GC-FID results represented in Table 3. Reaction conditions see S1.5.4 Product standards: **6** (black), (*rac*)-1-Phenylethanol (***rac*-7**, grey), and (*S*)-1-phenylethanol (***S*-7**, grey) were purchased from Alfa Aesar and diluted in EtOAc prior to injection.

Chiral phase GC-FID chromatograms from 4'-chloroacetophenone reduction

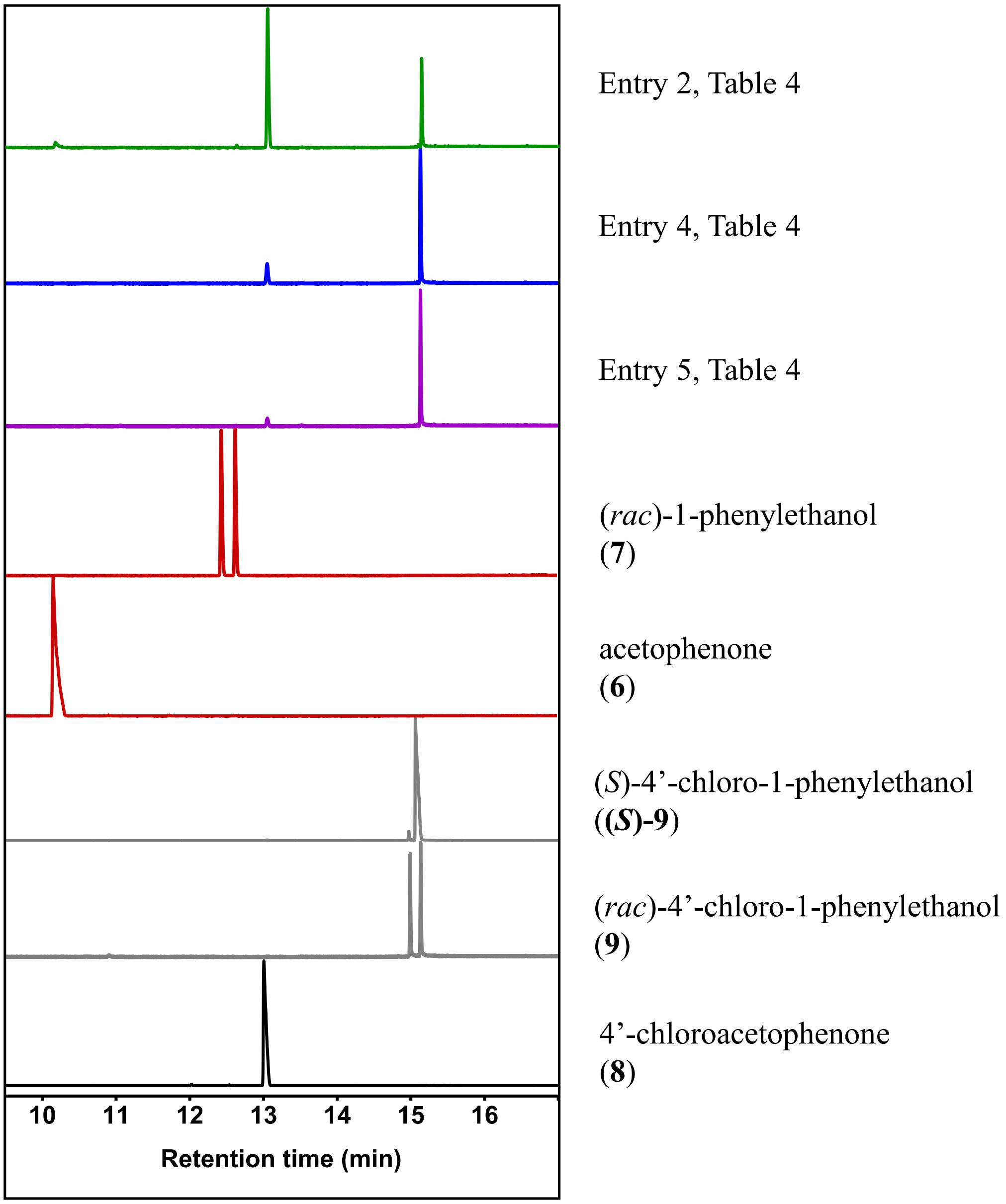

Figure S15. Examples of chiral-phase GC-FID results of 4'-chloroacetophenone (**8**) reductions in Table 4. Reaction conditions see S1.5.4, using 5 mM **8**. Product standards: **8** and (***S***)-**9** (available as 98%) were purchased from Alfa Aesar and diluted in EtOAc prior to injection. (*rac*)-**9** GC standard was prepared by mixing 1 mM **8** in water (1 vol% DMSO) with $NaBH_4$ and allowing to react overnight. A 50 µL aliquot was mixed well with 800 µL EtOAc, layers were separated, the organic layer was dried over Na_2SO_4 and taken for chiral GC-FID analysis. The potential products that can results from dehalogenation (**6** and **7**) are shown in red.

S2.12. Calculation of NADH-recycling simple E factor

E factor is a way to quantify waste relative to product in a reaction. Simple E factor does not count water nor a recyclable catalyst as waste.[8]

$$E\ Factor = \frac{\text{mass}_{\text{Tris - HCl}} + \text{mass}_{\text{ADH - 105}} + \text{mass}_{\text{NAD +}} + \text{mass}_{\text{unreacted } \mathbf{1}}}{\text{mass}_{\mathbf{(S)-2}}} \qquad \text{eq. S1}$$

S3. References

1. Reeve, H. A., Lauterbach, L., Lenz, O. & Vincent, K. A. Enzyme-Modified Particles for Selective Biocatalytic Hydrogenation by Hydrogen-Driven NADH Recycling. *ChemCatChem* **7**, 3480–3487 (2015).
2. Burgdorf, T., De Lacey, A. L. & Friedrich, B. Functional analysis by site-directed mutagenesis of the NAD^+-reducing hydrogenase from *Ralstonia eutropha*. *J. Bacteriol.* **184**, 6280–6288 (2002).
3. Lauterbach, L. & Lenz, O. Catalytic Production of Hydrogen Peroxide and Water by Oxygen-Tolerant [NiFe]-Hydrogenase during H_2 Cycling in the Presence of O_2 Scheme 1. Oxygen Species Produced upon Partial and Full Reduction of O_2. *J. Am. Chem. Soc.* **135**, 17897–17905 (2013).
4. Iranpoor, N. & Panahi, F. Nickel-catalyzed one-pot deoxygenation and reductive homocoupling of phenols via C-O activation using TCT reagent. *Org. Lett.* **17**, 214–217 (2015).
5. Roy, S. R., Sau, S. C. & Mandal, S. K. Chemoselective reduction of the carbonyl functionality through hydrosilylation: Integrating click catalysis with hydrosilylation in one pot. *J. Org. Chem.* **79**, 9150–9160 (2014).
6. Beaupre, B. A., Hoag, M. R., Roman, J., Holger Försterling, F. & Moran, G. R. Metabolic Function for Human Renalase: Oxidation of Isomeric Forms of β-NAD(P)H that Are Inhibitory to Primary Metabolism. *Biochemistry* **54**, 795–806 (2015).
7. Wang, X. & Yiu, H. H. P. Heterogeneous Catalysis Mediated Cofactor NADH Regeneration for Enzymatic Reduction. *ACS Catal.* **6**, 1880–1886 (2016).
8. Sheldon, R. A. The E factor 25 years on: the rise of green chemistry and sustainability. *Green Chem.* **19**, 18–43 (2017).
